# Effects of hypoxia-inducible factor prolyl hydroxylase inhibitors on transfusion and intravenous iron use in chronic kidney disease anemia: a systematic review and meta-analysis

**DOI:** 10.3389/fphar.2026.1916346

**Published:** 2026-09-16

**Authors:** Jiazheng Huang, Yixuan Wang, Huan Zhang, Awei Wang, Mianzhi Zhang, Yunsong Cao

**Affiliations:** 1 Dongfang Hospital, Beijing University of Chinese Medicine, Beijing, China; 2 Tianjin Hospital of Integrated Traditional Chinese and Western Medicine, Tianjin, China

**Keywords:** chronic kidney disease anemia, hypoxia-inducible factor prolyl hydroxylase inhibitors, intravenous iron, meta-analysis, red blood cell transfusion, roxadustat

## Abstract

**Background:**

Hypoxia-inducible factor prolyl hydroxylase inhibitors (HIF-PHIs) are novel oral agents for chronic kidney disease (CKD)-associated anemia. This study evaluated whether HIF-PHIs reduce red blood cell transfusion and intravenous iron exposure compared with placebo, standard care, or erythropoiesis-stimulating agents (ESAs) and assessed efficacy and safety.

**Methods:**

PubMed, Embase, and Web of Science were systematically searched. Randomized controlled trials comparing an HIF-PHI with placebo, standard care, or an ESA in adults with CKD-associated anemia were eligible. The primary outcomes were red blood cell transfusion and intravenous iron exposure. Secondary outcomes included changes in hemoglobin, cardiovascular events, and all-cause mortality, while safety was assessed using adverse event outcomes. Risk of bias was evaluated using the Cochrane Risk of Bias 2 tool. Furthermore, the certainty of evidence was assessed using the GRADE framework.

**Results:**

A total of 36 independent randomized controlled trials involving 27,680 participants with CKD-associated anemia were included. The trials evaluated six HIF-PHIs, namely, roxadustat, daprodustat, vadadustat, molidustat, enarodustat, and desidustat, against placebo, standard treatment or care, or an ESA. Compared with control interventions, HIF-PHIs significantly reduced the risk of any red blood cell transfusion (RR = 0.74, 95% CI: 0.58–0.93) and rescue red blood cell transfusion (RR = 0.70, 95% CI: 0.53–0.92). Intravenous iron outcomes generally favored HIF-PHIs, although neither the risk of any intravenous iron use (RR = 0.66, 95% CI: 0.38–1.14) nor the mean monthly intravenous iron dose (SMD = −0.19, 95% CI: −0.42 to 0.03) reached statistical significance, indicating residual uncertainty. Rates of major adverse cardiovascular events, all-cause mortality, and serious adverse events were not significantly increased with HIF-PHIs.

**Conclusion:**

In patients with CKD-associated anemia, HIF-PHIs improved hemoglobin without significantly increasing major adverse cardiovascular events, all-cause mortality, or serious adverse events; however, the small increase in any adverse event and the hyperkalemia signal in non-dialysis-dependent patients warrant attention. Together with the significant reduction in red blood cell transfusion and the possible reduction in intravenous iron use, these findings support HIF-PHIs as a promising oral treatment option. Longer follow-up and continued post-marketing surveillance are required to clarify long-term safety and effects across patient subgroups.

**Systematic Review Registration:**

https://www.crd.york.ac.uk/PROSPERO/view/CRD420261387833, Identifier CRD420261387833.

## 1 Introduction

Chronic kidney disease (CKD) is a major global public health concern, affecting approximately 10% of adults worldwide (Mazhar et al., 2023). Irrespective of the initiating cause, persistent nephron injury promotes maladaptive repair characterized by tubular atrophy, glomerulosclerosis, capillary rarefaction, interstitial inflammation, activation of matrix-producing myofibroblasts, and excessive extracellular matrix deposition, culminating in kidney fibrosis and progressive loss of renal function (Abbad et al., 2025; Liu, 2024; Liu H. et al., 2025). Key molecular drivers include transforming growth factor-β (TGF-β)/Smad signaling, oxidative stress and impaired Keap1-Nrf2 defenses, NOX4-dependent reactive oxygen species generation, NF-κB-mediated inflammation, and aryl hydrocarbon receptor signaling (Liu H. et al., 2025; Cao et al., 2022). In diabetic kidney disease, mesangial cell hypertrophy and proliferation, mesangial expansion, glomerular basement membrane thickening, podocyte injury, and tubulointerstitial damage further disrupt the glomerular filtration barrier and accelerate fibrosis (Feng et al., 2025).

Anemia is one of the most common systemic complications of CKD and becomes more prevalent as kidney function deteriorates. Its pathogenesis is multifactorial: loss and phenotypic transformation of renal erythropoietin-producing interstitial fibroblasts reduce endogenous erythropoietin production; inflammation and impaired renal clearance increase hepcidin, causing iron sequestration and reduced intestinal iron absorption; and uremic toxins, shortened erythrocyte survival, blood loss, nutritional deficiencies, and bone marrow hyporesponsiveness further impair erythropoiesis (Badura et al., 2024; Babitt and Lin, 2012). These interrelated abnormalities produce absolute or functional iron deficiency and resistance to erythropoietic stimulation. In addition to substantially impairing quality of life (National Kidney Foundation and Inc., 2006), CKD-associated anemia is independently associated with increased risks of cardiovascular events, hospitalization, and all-cause mortality (Palaka et al., 2020).

For decades, erythropoiesis-stimulating agents (ESAs), combined with intravenous or oral iron, have been the cornerstone of CKD-associated anemia treatment. Although these regimens effectively increase hemoglobin, important limitations remain in clinical practice: some patients have an inadequate response to ESAs (Drüeke and Massy, 2021), high ESA doses have been associated with increased cardiovascular risk (Sugahara et al., 2022), and maintaining target hemoglobin levels may require repeated intravenous iron administration or red blood cell transfusion (Willicombe and Roberts, 2024). Intravenous iron can cause hypersensitivity reactions and may increase infection risk (Shah et al., 2021; Van Doren et al., 2024), whereas red blood cell transfusion carries risks such as alloimmunization, hemolytic reactions, and iron overload (Linder et al., 2021). Accordingly, contemporary management of CKD-associated anemia aims not only to correct hemoglobin levels but also to reduce dependence on invasive, high-risk, and resource-intensive supportive interventions.

Hypoxia-inducible factor prolyl hydroxylase inhibitors (HIF-PHIs) provide a mechanistically distinct approach to CKD-associated anemia. By stabilizing hypoxia-inducible factor (HIF), these oral agents stimulate endogenous EPO production and improve iron availability by suppressing hepcidin and promoting iron absorption and mobilization (Li et al., 2025). Their efficacy may also be maintained in patients with inflammation-associated ESA hyporesponsiveness, potentially reducing transfusion requirements (Haase et al., 2026). Thus, beyond increasing hemoglobin, HIF-PHIs may directly reduce reliance on red blood cell transfusion and intravenous iron.

Several large-scale randomized controlled trials (RCTs) have evaluated the efficacy and safety of roxadustat, daprodustat, vadadustat, and other HIF-PHIs in patients with CKD-associated anemia who are receiving or not receiving dialysis (Fishbane et al., 2022; Coyne et al., 2022; Koury et al., 2022). Secondary studies of HIF-PHIs have also gradually emerged. A Cochrane systematic review comprehensively assessed the benefits and potential harms of HIF stabilizers for the management of CKD anemia (Natale et al., 2022); another systematic review and meta-analysis focused on the effects of HIF-PHIs on cardiac and kidney-related adverse events in patients with non-dialysis CKD; and other reviews have compared the relative efficacy and safety of different HIF-PHIs in improving hemoglobin, iron-metabolism parameters, major adverse cardiovascular events (MACE), and all-cause mortality (Zheng et al., 2023; Singh et al., 2021a). However, whether HIF-PHIs can substantially reduce “red blood cell transfusion” and “intravenous iron use”—two key hard endpoints directly related to patients’ clinical risk, treatment burden, and healthcare resource consumption—remains uncertain. Although some original studies suggest that HIF-PHIs may reduce transfusion dependence by optimizing endogenous EPO production and iron metabolism (Ikeda, 2021; Toka et al., 2025), results from individual RCTs remain inconsistent, and a dedicated and comprehensive systematic synthesis is lacking.

We, therefore, conducted a systematic review and meta-analysis of all available RCT evidence to quantify the efficacy and safety of HIF-PHIs, compared with placebo, standard care, or ESAs, in reducing red blood cell transfusion and intravenous iron requirements in adults with CKD-associated anemia.

## 2 Methods

### 2.1 Data sources and search strategy

PubMed, Embase, and Web of Science were systematically searched from database inception through 10 April 2026. The strategy combined four core concepts—chronic kidney disease, anemia, HIF-PHIs, and randomized controlled trials—with OR expansions for individual drug names and compound codes; complete search strings and line-by-line retrieval counts are provided in Supplementary Table S1.

### 2.2 Study selection and eligibility criteria

Prespecified eligibility criteria were as follows: (1) adults with CKD-associated anemia, including dialysis-dependent CKD (DD-CKD) and non-dialysis-dependent CKD (NDD-CKD); (2) treatment with any HIF-PHI used for CKD-associated anemia, including but not limited to roxadustat, daprodustat, vadadustat, molidustat, enarodustat, and desidustat; (3) a comparator of placebo, usual treatment or care, or an ESA such as epoetin alfa, darbepoetin alfa, or methoxy polyethylene glycol-epoetin beta; and (4) reporting of at least one outcome of interest. We excluded studies in which anemia was attributable to a cause other than CKD, studies comparing only different doses of the same HIF-PHI, studies for which the full text or essential data could not be obtained, and studies that did not report an extractable outcome of interest. The primary outcomes were any red blood cell transfusion and any intravenous iron exposure during the randomized treatment or follow-up period. Key secondary outcomes included mean monthly intravenous iron dose, rescue red blood cell transfusion, changes in hemoglobin and iron indices, all-cause mortality, MACE, serious adverse events (SAEs), and treatment discontinuation due to adverse events. Only studies published in English were eligible. The review was registered in PROSPERO (CRD420261387833).

Two reviewers (JH and YW) independently screened the retrieved records. Titles and abstracts were assessed against the prespecified eligibility criteria, and the full texts of potentially eligible reports were subsequently reviewed. Disagreements were resolved through discussion or adjudication by a third reviewer (YD). The study-selection process is shown in Figure 1.

**FIGURE 1:**
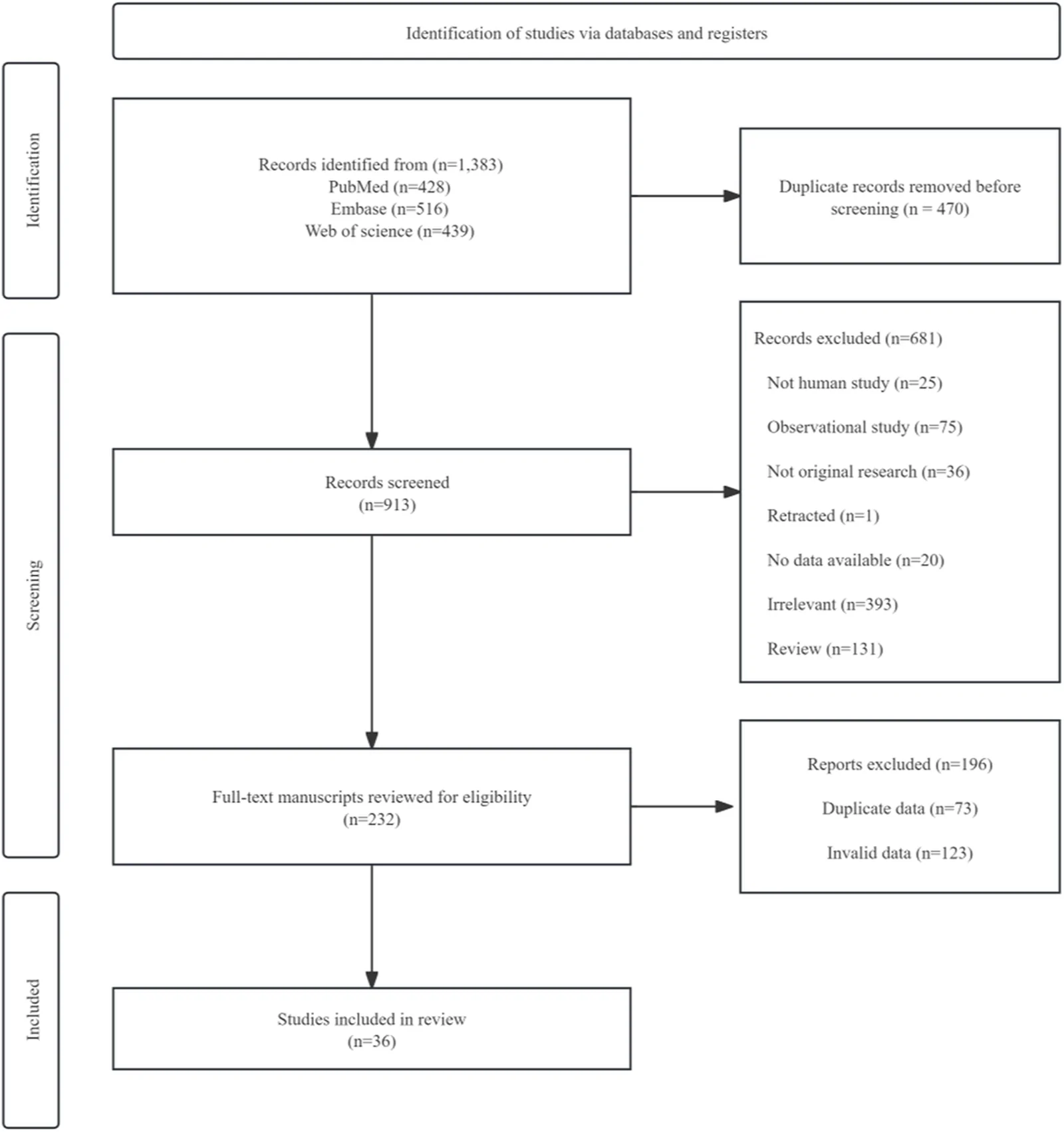
Flow diagram of the literature search and study-selection process.

### 2.3 Data extraction

Two authors (JH and YW) independently extracted data from the eligible RCTs using a standardized procedure and cross-checked the completed forms. When essential information was missing or unclear, the corresponding study authors or sponsors were contacted by email for additional data. The prespecified extraction form captured the first author, publication year, trial registration number, study design, dialysis status, intervention and comparator, sample size, follow-up duration, baseline hemoglobin and iron indices, and outcome data on red blood cell transfusion, intravenous iron use, hemoglobin, iron metabolism, and safety.

### 2.4 Outcome definitions

The primary outcomes were (1) red blood cell transfusion during treatment, defined as the proportion of participants who received at least one red blood cell transfusion, and (2) intravenous iron exposure, defined as the proportion who received at least one intravenous iron treatment. Treatment acceptability was evaluated using the reported rates of treatment discontinuation, any adverse event (AE), and serious adverse events (SAEs).

Secondary outcomes were (1) rescue red blood cell transfusion, defined as the proportion of participants who received transfusion because of inadequate anemia control or fulfillment of prespecified rescue criteria; (2) intravenous iron dose, defined as the mean monthly dose during the study; (3) hemoglobin outcomes, including change from baseline and hemoglobin during the maintenance period; and (4) changes in iron indices, including ferritin and transferrin saturation. Additional safety outcomes included all-cause mortality, MACE, treatment discontinuation due to adverse events, and other adverse events reported by the included studies.

### 2.5 Risk of bias and certainty of evidence

Outcome-level risk of bias was assessed using the Cochrane Risk of Bias 2 (RoB 2) tool. The domains comprised the randomization process, deviations from intended interventions, missing outcome data, outcome measurement, and selection of the reported result. Two reviewers (JH and YW) independently completed the assessments, with disagreements resolved through discussion with a third reviewer (YD).

Certainty of evidence was assessed using the GRADE approach, with potential downgrading for risk of bias, inconsistency, indirectness, imprecision, and publication bias. Certainty was rated for the primary and key secondary outcomes.

### 2.6 Statistical analysis

Analyses were conducted using the meta and metafor packages in R version 4.5.1. Unless otherwise specified, statistical significance was defined as a two-sided *p*-value < 0.05. Dichotomous outcomes were pooled as risk ratios (RRs) with 95% confidence intervals (CIs); continuous outcomes were summarized as mean differences (MDs) when units and reporting scales were comparable and as standardized mean differences (SMDs) when units, assessment windows, or reporting scales differed across studies. Primary analyses used DerSimonian–Laird random-effects inverse-variance models to account for anticipated clinical and methodological heterogeneity. Between-study heterogeneity was assessed using Cochran’s Q, I^2^, and τ^2^; I^2^ < 30% was considered low, 30%–60% moderate, and >60% substantial heterogeneity. Prespecified subgroup analyses examined dialysis status, comparator type, and HIF-PHI; pooled estimates were reported only when a subgroup contained at least two independent studies or eligible comparisons. Sensitivity analyses used fixed-effect models, leave-one-out analyses, and restriction to studies at low risk of bias. For outcomes reported by at least 10 studies, funnel plots were used to assess publication bias and small-study effects, supplemented by Egger’s or Peters’ test, as appropriate for the outcome type. Continuous outcomes with substantial heterogeneity were interpreted with consideration of effect direction, confidence intervals, subgroup analyses, and sensitivity analyses.

### 2.7 Role of the funding source

The funder had no role in study design, data collection, analysis, interpretation, manuscript preparation, or the decision to submit the work.

## 3 Results

### 3.1 Study characteristics

The review included 36 independent RCTs involving 27,680 participants and published between 2016 and 2025; one related subgroup report was retained as Supplementary Material but was not counted as an independent RCT in the primary analysis and is listed separately in Supplementary Table S2. After multi-arm trials and stratified reports were separated according to prespecified rules, 43 analyzable comparisons of HIF-PHIs versus controls were available. The handling of multi-arm trials and shared control groups is detailed in Supplementary Table S2. A total of six HIF-PHIs were represented: roxadustat (n = 14 comparisons), vadadustat (n = 11), daprodustat (n = 8), molidustat (n = 6), desidustat (n = 2), and enarodustat (n = 2). Comparators were darbepoetin alfa in 18 comparisons, placebo in 13, epoetin alfa or a related agent (including CERA, MPG-EPO, and rhEPO) in 11, and mixed ESA-based standard care in one. The study populations included both NDD-CKD and DD-CKD, with the latter comprising maintenance hemodialysis, peritoneal dialysis, and incident dialysis populations. Studies were conducted in Asia, North America, Europe, and multinational settings, with a substantial proportion of single-country studies from Asia. Individual sample sizes ranged from 51 to 3,872 participants, mean ages ranged from approximately 48.0–72.0 years, and follow-up ranged from 6 weeks to 4 years, although most efficacy assessments were conducted at 24–52 weeks. Several included phase 3 programs also contributed longer safety observation: selected roxadustat trials followed participants for 104–208 weeks, and ASCEND-ND had a median cardiovascular follow-up of 1.9 years (Pergola et al., 2016; Chertow et al., 2021; Johansen et al., 2023; Kooienga et al., 2024; Eckardt et al., 2021; Csiky et al., 2021). In addition, the molidustat DIALOGUE 3 and 5 open-label extension studies followed selected participants from the parent trials for up to 36 months; because these cohorts consisted of trial completers and were not independent randomized comparisons, they were considered supportive rather than pooled as separate RCTs (Ak et al., 2019). Characteristics of the included studies are summarized in Table 1.

**TABLE 1:** Characteristics of the 36 included randomized controlled trials.

Source	Country	Population	Participants, no. (Sex, no. [%])	Age, mean (SD), y	HIF-PHI drug	Comparator	Sample size (HIF-PHI/control)	Regimen	Duration (wk)
Nangaku et al. (2021a)	Japan	NDD-CKD + DD-CKD	109 (69 male [63%]; 40 female [37%])	66.9 (11.1)	Vadadustat	Placebo	81/28	150/300/600 mg QD	16
Pergola et al. (2016)	NR	NDD-CKD	210 (95 male [45%]; 115 female [55%])	66.4 (10.8)	Vadadustat	Placebo	138/72	450 mg QD	20
Toka et al. (2025)	United States	DD-CKD	456 (262 male [57%]; 194 female [43%])	60.9 (13.5)	Vadadustat	MPG-EPO	304/152	600/900 mg TIW	52
Chertow et al. (2021)	International	NDD-CKD	3,476 (1,539 male [44%]; 1937 female [56%])	66.0 (13.7)	Vadadustat	Darbepoetin alfa	1741/1735	300 mg QD	52
Coyne et al. (2022)	Global	HD	407 (229 male [56%]; 178 female [44%])	NR	Daprodustat	Epoetin alfa	270/137	Dose based on prior ESA	52
Johansen et al. (2023)	Global	NDD-CKD	614 (261 male [43%]; 353 female [57%])	NR	Daprodustat	Placebo	307/307	2/4 mg QD	28
Kooienga et al. (2024)	United States/Europe	DD-CKD	319 (183 male [57%]; 136 female [43%])	61.0 (12.9)	Vadadustat	Darbepoetin alfa	105/108; 106/108	300/450 mg QD; 600/750 mg TIW	52
Eckardt et al. (2021)	International	DD-CKD	3,923 (2,214 male [56%]; 1709 female [44%])	58.0 (13.9)	Vadadustat	Darbepoetin alfa	1958/1965	300 mg QD	52
Fishbane et al. (2022)	International	DD-CKD	2,133 (1,251 male [59%]; 882 female [41%])	54.0 (15.2)	Roxadustat	Epoetin alfa	1,068/1,065	Dose based on prior ESA/weight	208
Akizawa et al. (2019)	Japan	NDD-CKD	107 (50 male [47%]; 57 female [53%])	63.8 (9.2)	Roxadustat	Placebo	80/27	50/70/100 mg TIW or QW	24
Hou et al. (2022)	China	PD	129 (72 male [56%]; 57 female [44%])	48.1 (12.3)	Roxadustat	ESAs	86/43	100/120 mg by weight	24
Fishbane et al. (2021)	Global	NDD-CKD	2,781 (1,167 male [42%]; 1,614 female [58%])	61.6 (14.4)	Roxadustat	Placebo	1,393/1,388	70 mg TIW	208
Shutov et al. (2021)	Mostly Europe	NDD-CKD	594 (268 male [45%]; 326 female [55%])	NR	Roxadustat	Placebo	391/203	70/100 mg TIW by weight	104
Barratt et al. (2021)	Europe	NDD-CKD	616 (274 male [44%]; 342 female [56%])	66.3 (14.0)	Roxadustat	Darbepoetin alfa	323/293	70/100 mg TIW by weight	104
Csiky et al. (2021)	Europe	DD-CKD	836 (480 male [57%]; 356 female [43%])	61.4 (13.6)	Roxadustat	ESA	415/421	Dose based on prior ESA	104
Coyne et al. (2021)	United States	NDD-CKD	922 (371 male [40%]; 551 female [60%])	64.9 (12.8)	Roxadustat	Placebo	616/306	70/100 mg TIW by weight	208
Chen et al. (2019)	China	NDD-CKD	152 (56 male [37%]; 96 female [63%])	54.2 (13.2)	Roxadustat	Placebo	101/51	70/100 mg TIW by weight	8
Provenzan et al. (2021)	International	DD-CKD	1,043 (616 male [59%]; 427 female [41%])	54.0 (14.6)	Roxadustat	Epoetin alfa	522/521	70/100 mg TIW by weight	52
Provenzano et al. (2016)	NR	HD	90 (59 male [66%]; 31 female [34%])	56.9 (11.9)	Roxadustat	Epoetin alfa	67/23	1.0–2.0 mg/kg TIW	19
Agrawal et al. (2022)	India/Sri Lanka	NDD-CKD	588 (296 male [50%]; 292 female [50%])	52.8 (13.8)	Desidustat	Darbepoetin alfa	294/294	100 mg TIW	24
Gang et al. (2022)	India	DD-CKD	392 (269 male [69%]; 123 female [31%])	51.0 (13.7)	Desidustat	Epoetin alfa	196/196	100/125/150 mg TIW	24
Singh et al. (2021b)	Global	NDD-CKD	3,872 (1,699 male [44%]; 2,173 female [56%])	NR	Daprodustat	Darbepoetin alfa	1937/1935	1–24 mg QD	52
Holdstock et al. (2019)	Global	NDD-CKD	235 (97 male [41%]; 138 female [59%])	66.1 (13.0)	Daprodustat	rhEPO standard of care	156/79	1/2/4 mg QD; 2 mg QD	24
Meadowcroft et al. (2019)	Global	HD	210 (134 male [64%]; 76 female [36%])	59.6 (14.4)	Daprodustat	rhEPO (after 4-week placebo)	171/39	4/6/8/10/12 mg QD	24
Nangaku et al. (2021b)	Japan	NDD-CKD	217 (135 male [62%]; 82 female [38%])	70.0 (10.1)	Daprodustat	Epoetin beta pegol (CERA)	108/109	2/4 mg QD	52
Charytan et al. (2021)	United States	HD	741 (402 male [54%]; 339 female [46%])	58.0 (13.4)	Roxadustat	Epoetin alfa	370/371	70/100/150/200 mg TIW	52
Akizawa et al. (2021a)	Japan	HD	172 (122 male [71%]; 50 female [29%])	64.0 (10.6)	Enarodustat	Darbepoetin alfa	86/86	4 mg QD	24
Nangaku et al. (2021c)	Japan	NDD-CKD	304 (148 male [49%]; 156 female [51%])	72.0 (9.9)	Vadadustat	Darbepoetin alfa	151/153	150–600 mg QD	52
Chen et al. (2017)	China	NDD-CKD + DD-CKD	187 (83 male [44%]; 104 female [56%])	49.8 (14.0)	Roxadustat (FG-4592)	Placebo/epoetin alfa	135/52	1.1–2.3 mg/kg TIW	6–8
Yamamoto et al. (2021a)	Japan	NDD-CKD	164 (99 male [60%]; 65 female [40%])	70.7 (10.4)	Molidustat	Darbepoetin alfa	82/82	25/50 mg QD	52
Akizawa et al. (2021b)	Japan	HD	229 (140 male [61%]; 89 female [39%])	65.7 (10.4)	Molidustat	Darbepoetin alfa	153/76	75 mg QD	52
Liang et al. (2024)	China	NDD-CKD	155 (62 male [40%]; 93 female [60%])	54.3 (13.0)	Enarodustat	Placebo	103/52	1/2/4/6/8 mg QD	24
Yamamoto et al. (2021b)	Japan	NDD-CKD	162 (100 male [62%]; 62 female [38%])	71.7 (9.7)	Molidustat	Darbepoetin alfa	82/80	25 mg QD	52
Singh et al. (2022)	Global	DD-CKD	312 (194 male [62%]; 118 female [38%])	NR	Daprodustat	Darbepoetin alfa	157/155	Dose based on Hb	52
Akizawa et al. (2020)	Japan	HD	271 (180 male [66%]; 91 female [34%])	64.0 (10.5)	Daprodustat	Darbepoetin alfa	136/135	1–24 mg QD	52
Macdougall et al. (2019)	Europe/Asia-Pacific/United States/Japan	NDD-CKD + DD-CKD	444 (248 male [56%]; 196 female [44%])	64.2 (12.8)	Molidustat	Placebo/darbepoetin/epoetinα/β	350/94	25/50/75/150 mg QD; 25/50 mg BID	16

DD-CKD, dialysis-dependent chronic kidney disease; NDD-CKD, non-dialysis-dependent chronic kidney disease; HD, hemodialysis; PD, peritoneal dialysis; ESA, erythropoiesis-stimulating agent; MPG-EPO, methoxy polyethylene glycol-epoetin beta; CERA, continuous erythropoietin receptor activator; rhEPO, recombinant human erythropoietin; Hb, hemoglobin; BID, twice daily; QD, once daily; QW, once weekly; TIW, three times weekly; No., number; NR, not reported. Regimens refer to HIF-PHI dosing only. Duration is reported in weeks; durations reported in years were converted using 52 weeks per year. Age values are the overall mean (SD) in years; when not reported directly, they were calculated from group-level means, SDs, and sample sizes. Studies reporting age only as a median, range, or interquartile range were recorded as NR. Participant and sex data were extracted from the delivery package and complete PDF reports; when sex was reported only as a percentage, counts were calculated from the reported denominator and percentage.

### 3.2 HIF-PHIs reduced the risk of any red blood cell transfusion

A random-effects meta-analysis of 12 eligible comparisons (Fishbane et al., 2022; Coyne et al., 2022; Barratt et al., 2021; Csiky et al., 2021; Coyne et al., 2021; Provenzan et al., 2021; Holdstock et al., 2019; Meadowcroft et al., 2019; Charytan et al., 2021; Yamamoto et al., 2021a; Yamamoto et al., 2021b; Singh et al., 2022) showed that HIF-PHIs significantly reduced the risk of any red blood cell transfusion compared with control interventions, which were predominantly ESAs but also included placebo (RR = 0.74, 95% CI: 0.58–0.93; I^2^ = 54.0%; Figure 2). After stratification by dialysis status, the reduction was significant in DD-CKD (Fishbane et al., 2022; Coyne et al., 2022; Csiky et al., 2021; Provenzan et al., 2021; Meadowcroft et al., 2019; Charytan et al., 2021; Singh et al., 2022) (RR = 0.74, 95% CI: 0.64–0.86; I^2^ = 0%), whereas NDD-CKD showed a non-significant trend toward benefit (Barratt et al., 2021; Coyne et al., 2021; Holdstock et al., 2019; Yamamoto et al., 2021a; Yamamoto et al., 2021b) (RR = 0.72, 95% CI: 0.29–1.83; I^2^ = 77.4%). By individual drug, the risk was significantly reduced with roxadustat (Fishbane et al., 2022; Barratt et al., 2021; Csiky et al., 2021; Coyne et al., 2021; Provenzan et al., 2021; Charytan et al., 2021) (RR = 0.73, 95% CI: 0.54–0.99), while daprodustat (Coyne et al., 2022; Holdstock et al., 2019; Meadowcroft et al., 2019; Singh et al., 2022) (RR = 0.80, 95% CI: 0.54–1.20) and molidustat (Yamamoto et al., 2021a; Yamamoto et al., 2021b) (RR = 0.35, 95% CI: 0.05–2.25) showed non-significant reductions. Overall, HIF-PHIs reduced the risk of red blood cell transfusion, with the clearest effects observed in DD-CKD and roxadustat trials. Complete subgroup estimates are provided in Supplementary Tables S5 and S6, and the drug-stratified forest plot is shown in Supplementary Figure S1.

**FIGURE 2:**
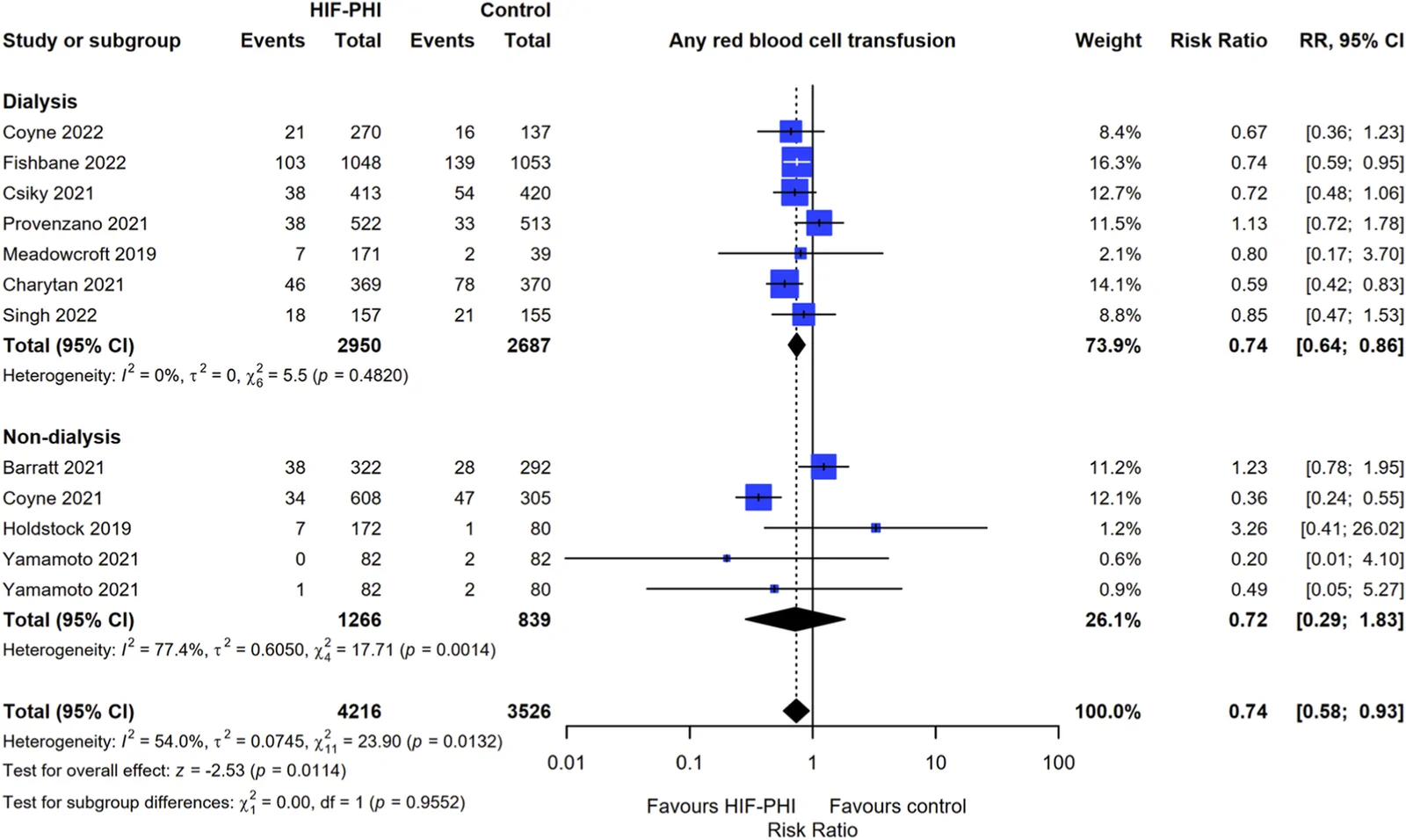
Meta-analysis of HIF-PHIs and any red blood cell transfusion in chronic kidney disease anemia.

### 3.3 HIF-PHIs reduced the need for rescue red blood cell transfusion

Meta-analysis of 9 eligible comparisons (Fishbane et al., 2022; Nangaku et al., 2021a; Pergola et al., 2016; Fishbane et al., 2021; Barratt et al., 2021; Csiky et al., 2021; Chen et al., 2017; Yamamoto et al., 2021a; Yamamoto et al., 2021b) showed that HIF-PHIs significantly reduced the risk of rescue red blood cell transfusion (RR = 0.70, 95% CI: 0.53–0.92; I^2^ = 53.1%; Figure 3). In drug-specific analyses, rescue transfusion was significantly reduced with roxadustat (Fishbane et al., 2022; Fishbane et al., 2021; Barratt et al., 2021; Csiky et al., 2021; Chen et al., 2017) (RR = 0.73, 95% CI: 0.54–0.97) and vadadustat (Nangaku et al., 2021a; Pergola et al., 2016) (RR = 0.13, 95% CI: 0.02–0.76), whereas molidustat showed a non-significant reduction (Yamamoto et al., 2021a; Yamamoto et al., 2021b) (RR = 0.62, 95% CI: 0.08–4.95). The direction of effect was generally consistent across drug subgroups, supporting a lower need for rescue transfusion with HIF-PHIs. The drug-stratified forest plot is provided in Supplementary Figure S2.

**FIGURE 3:**
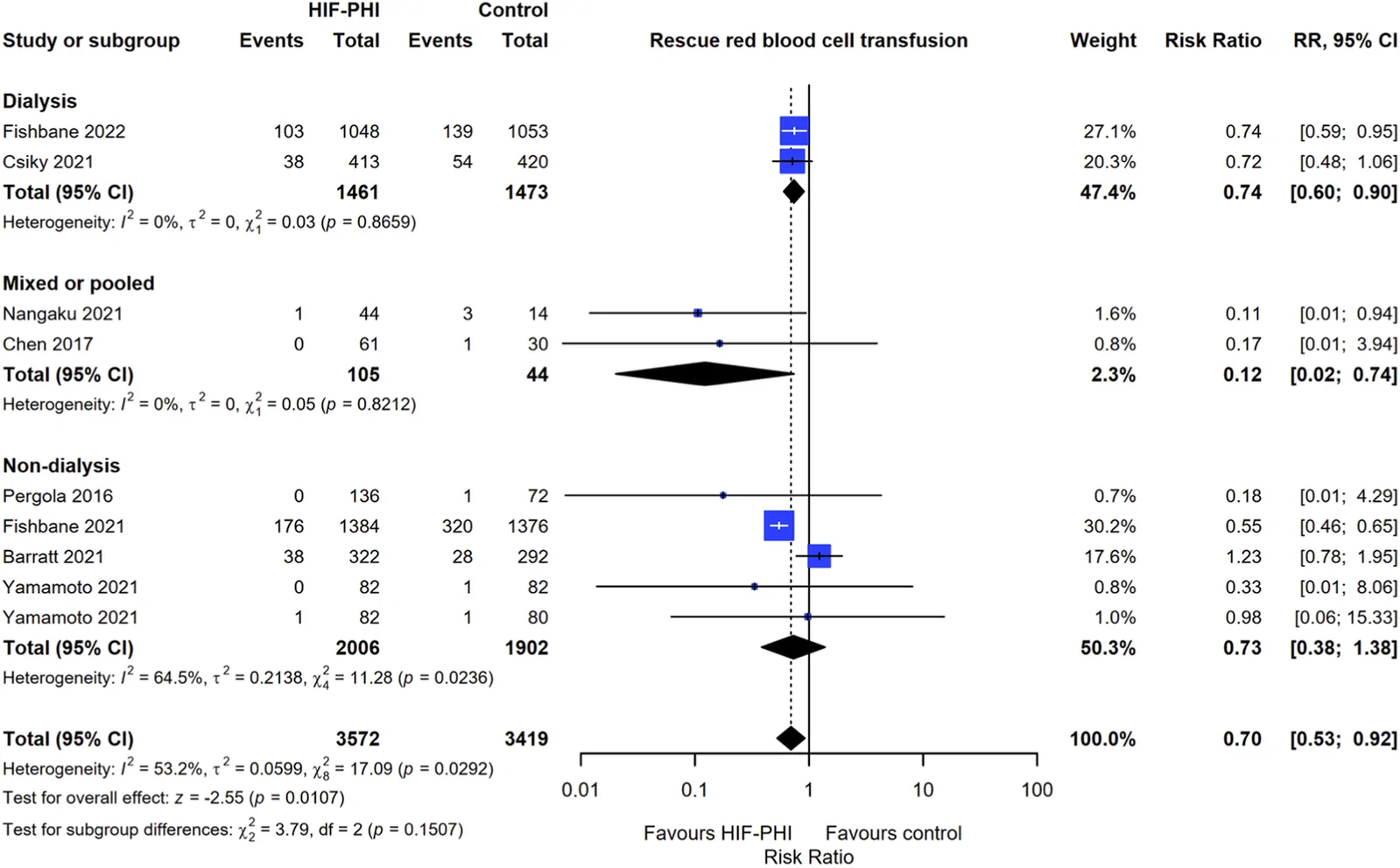
Meta-analysis of HIF-PHIs and rescue red blood cell transfusion in chronic kidney disease anemia.

### 3.4 HIF-PHIs showed an overall trend toward reduced intravenous iron use

A total of five RCTs (Barratt et al., 2021; Csiky et al., 2021; Nangaku et al., 2021b; Yamamoto et al., 2021a; Akizawa et al., 2021b) reported any intravenous iron use. The random-effects meta-analysis favored HIF-PHIs, but the difference was not statistically significant (RR = 0.66, 95% CI: 0.38–1.14; I^2^ = 87.3%; Figure 4). By dialysis status, HIF-PHIs significantly reduced intravenous iron use in NDD-CKD (Barratt et al., 2021; Nangaku et al., 2021b; Yamamoto et al., 2021a) (RR = 0.54, 95% CI: 0.33–0.88; I^2^ = 0%), whereas the reduction in DD-CKD was not significant (Csiky et al., 2021; Akizawa et al., 2021b) (RR = 0.66, 95% CI: 0.31–1.43; I^2^ = 96.6%). By drug, roxadustat significantly reduced intravenous iron use (Barratt et al., 2021; Csiky et al., 2021) (RR = 0.45, 95% CI: 0.38–0.54; I^2^ = 0%), whereas molidustat showed no significant difference (Yamamoto et al., 2021a; Akizawa et al., 2021b) (RR = 0.98, 95% CI: 0.80–1.21; I^2^ = 0%). For the mean monthly intravenous iron dose, the pooled result from five RCTs (Fishbane et al., 2022; Barratt et al., 2021; Csiky et al., 2021; Yamamoto et al., 2021a; Akizawa et al., 2021b) favored HIF-PHIs but did not reach statistical significance (SMD = −0.19, 95% CI: −0.42 to 0.03; I^2^ = 89.2%). Thus, both intravenous iron use and monthly dose showed an overall reduction, with clearer effects in NDD-CKD and roxadustat subgroups. The supplementary forest plot is provided in Supplementary Figure S3, and drug-stratified plots are shown in Supplementary Figures S4 and S5.

**FIGURE 4:**
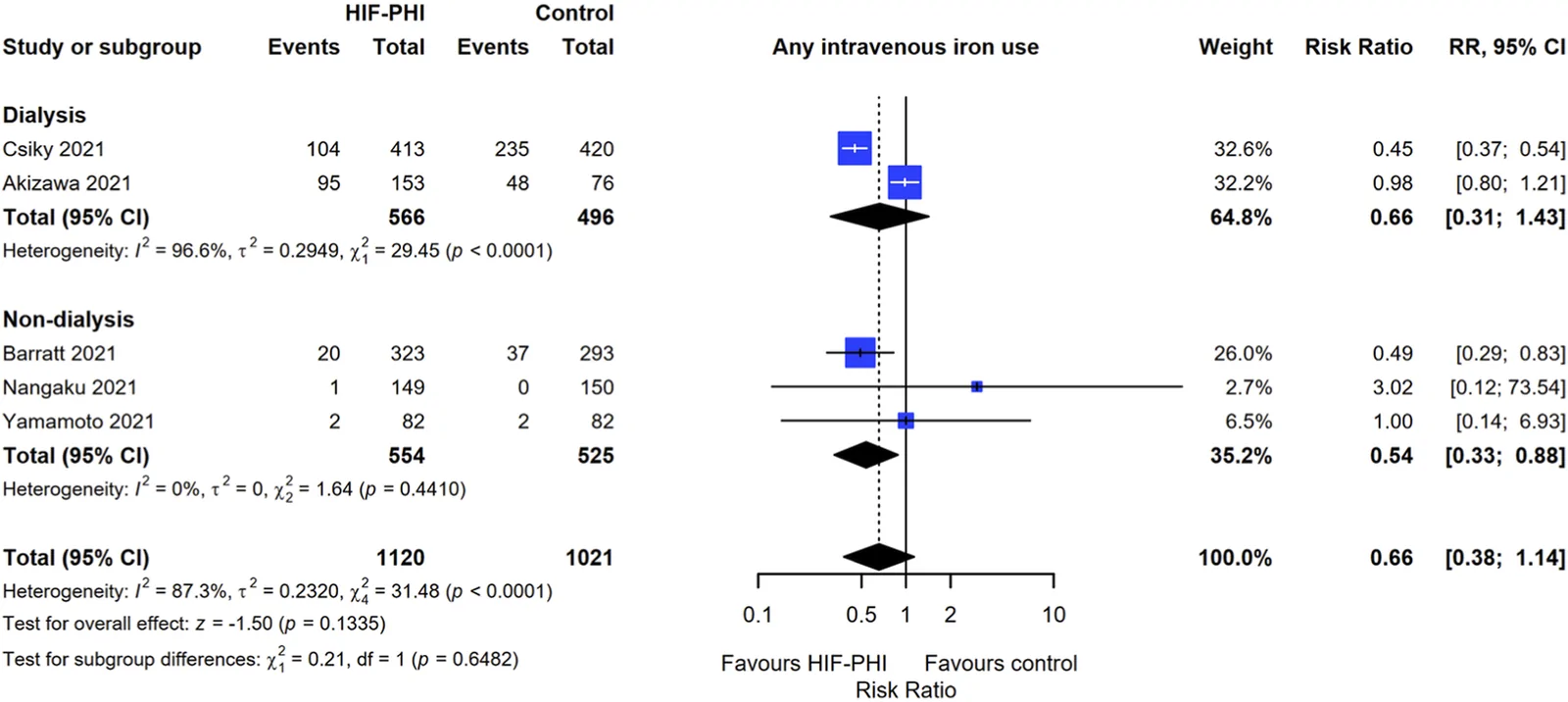
Meta-analysis of HIF-PHIs and any intravenous iron use in chronic kidney disease anemia.

### 3.5 Hemoglobin increased without evidence of excessive correction

A total of 9 comparisons (Akizawa et al., 2019; Csiky et al., 2021; Coyne et al., 2021; Chen et al., 2019; Provenzan et al., 2021; Charytan et al., 2021; Akizawa et al., 2021a; Liang et al., 2024) reported a change in hemoglobin from baseline. HIF-PHIs significantly increased hemoglobin in the random-effects meta-analysis (MD = 0.99 g/dL, 95% CI: 0.45–1.52; I^2^ = 98.7%); the corresponding forest plot is shown in Supplementary Figure S6. The increase was greater in NDD-CKD (Akizawa et al., 2019; Coyne et al., 2021; Chen et al., 2019; Liang et al., 2024) (MD = 1.65 g/dL, 95% CI: 1.30–2.01; I^2^ = 88.9%) but remained significant in DD-CKD (Csiky et al., 2021; Provenzan et al., 2021; Charytan et al., 2021; Akizawa et al., 2021a) (MD = 0.20 g/dL, 95% CI: 0.09–0.30; I^2^ = 49.7%). A total of 3 eligible comparisons (Coyne et al., 2022; Kooienga et al., 2024) reported maintenance-period or average hemoglobin, for which the difference between HIF-PHIs and controls was not statistically significant (MD = −0.27 g/dL, 95% CI: −0.56 to 0.02; I^2^ = 79.7%); the corresponding forest plot is shown in Supplementary Figure S7. For iron indices, 7 comparisons (Akizawa et al., 2019; Hou et al., 2022; Barratt et al., 2021; Coyne et al., 2021; Chen et al., 2019; Provenzano et al., 2016; Meadowcroft et al., 2019) reported change in ferritin from baseline; the pooled estimate favored a reduction with HIF-PHIs but was not statistically significant (SMD = −0.14, 95% CI: −0.28 to 0.01; I^2^ = 42.7%), as shown in Supplementary Figure S8. Overall, HIF-PHIs increased hemoglobin during treatment without producing higher maintenance-period levels than those observed with controls; descriptive analyses of highly heterogeneous continuous outcomes are provided in Supplementary Table S10.

### 3.6 MACE, all-cause mortality, and serious adverse events were not significantly increased

A total of 17 eligible comparisons (Fishbane et al., 2022; Coyne et al., 2022; Chertow et al., 2021; Johansen et al., 2023; Kooienga et al., 2024; Eckardt et al., 2021; Fishbane et al., 2021; Barratt et al., 2021; Provenzano et al., 2016; Singh et al., 2021b; Holdstock et al., 2019; Meadowcroft et al., 2019; Akizawa et al., 2021b; Singh et al., 2022) reported MACE. The random-effects meta-analysis showed no significant difference between HIF-PHIs and controls (RR = 1.01, 95% CI: 0.95–1.06; I^2^ = 0%; Figure 5). A total of 18 eligible comparisons (Fishbane et al., 2022; Pergola et al., 2016; Kooienga et al., 2024; Hou et al., 2022; Fishbane et al., 2021; Shutov et al., 2021; Barratt et al., 2021; Csiky et al., 2021; Provenzano et al., 2016; Agrawal et al., 2022; Gang et al., 2022; Singh et al., 2021b; Holdstock et al., 2019; Meadowcroft et al., 2019; Yamamoto et al., 2021a; Yamamoto et al., 2021b; Singh et al., 2022) reported all-cause mortality during treatment, and the pooled estimate did not indicate an increased risk with HIF-PHIs (RR = 1.05, 95% CI: 0.96–1.14; I^2^ = 0%). A total of 19 eligible comparisons (Fishbane et al., 2022; Kooienga et al., 2024; Eckardt et al., 2021; Akizawa et al., 2019; Hou et al., 2022; Fishbane et al., 2021; Shutov et al., 2021; Barratt et al., 2021; Csiky et al., 2021; Chen et al., 2019; Agrawal et al., 2022; Gang et al., 2022; Singh et al., 2021b; Holdstock et al., 2019; Akizawa et al., 2021a; Akizawa et al., 2021b; Singh et al., 2022) reported SAEs, with no statistically significant difference between groups (RR = 1.04, 95% CI: 0.99–1.10; I^2^ = 47.3%). For any adverse event, 18 RCTs (Fishbane et al., 2022; Akizawa et al., 2019; Hou et al., 2022; Fishbane et al., 2021; Shutov et al., 2021; Barratt et al., 2021; Csiky et al., 2021; Coyne et al., 2021; Chen et al., 2019; Provenzan et al., 2021; Agrawal et al., 2022; Gang et al., 2022; Singh et al., 2021b; Holdstock et al., 2019; Meadowcroft et al., 2019; Akizawa et al., 2021a; Akizawa et al., 2021b; Singh et al., 2022) showed a small increase with HIF-PHIs (RR = 1.02, 95% CI: 1.003–1.031; I^2^ = 0%; Supplementary Figure S9). A total of 5 RCTs (Hou et al., 2022; Shutov et al., 2021; Coyne et al., 2021; Chen et al., 2019; Provenzan et al., 2021) reported hyperkalemia, with no significant overall difference (RR = 1.21, 95% CI: 0.86–1.70; I^2^ = 37.0%; Supplementary Figure S10); however, subgroup analysis showed an increased risk in NDD-CKD (Shutov et al., 2021; Coyne et al., 2021; Chen et al., 2019) (RR = 1.39, 95% CI: 1.05–1.83; I^2^ = 0%) but not in DD-CKD (Hou et al., 2022; Provenzan et al., 2021) (RR = 0.93, 95% CI: 0.39–2.22; I^2^ = 38.3%). All five trials contributing data on hyperkalemia evaluated roxadustat, and the NDD-CKD estimate was derived from three placebo-controlled roxadustat trials; therefore, comparative estimates for other HIF-PHIs were not available. Overall, HIF-PHIs did not significantly increase MACE, all-cause mortality, or SAEs, although any adverse events were slightly more frequent and a hyperkalemia signal was observed in NDD-CKD; complete safety estimates are provided in Supplementary Table S7.

**FIGURE 5:**
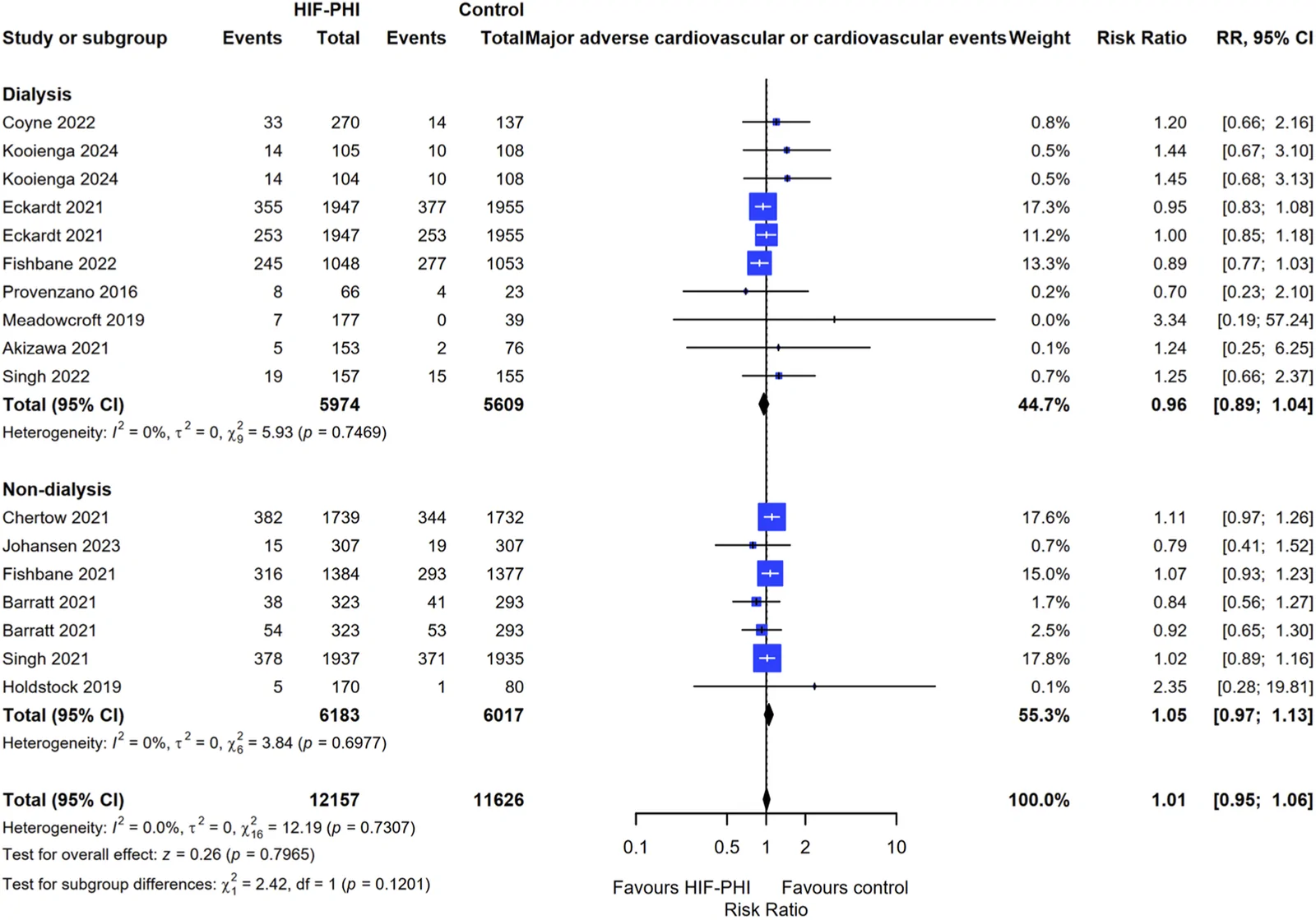
Meta-analysis of HIF-PHIs and major adverse cardiovascular events in chronic kidney disease anemia.

### 3.7 Risk of bias, publication bias, and sensitivity analyses

The outcome-level RoB 2 assessment comprised 31 key outcome evaluations: 3 were judged to be at low risk of bias and 28 to raise some concerns, with none at high risk. Concerns were driven mainly by open-label designs, in which decisions regarding transfusion or intravenous iron could be influenced by clinician judgment and differences in study protocols or treatment management. The pooled effects for any and rescue red blood cell transfusion remained directionally consistent in fixed-effect models and leave-one-out analyses, and the rescue red blood cell transfusion effect remained directionally consistent when restricted to studies at low risk of bias, whereas intravenous iron outcomes were more sensitive to heterogeneity, open-label treatment practices, and individual studies. Funnel plots and tests for small-study effects did not indicate clear publication bias. Detailed findings are reported in Supplementary Tables S3, S4, and S7–S10 and Supplementary Figures S11–S15.

Certainty of evidence was assessed using the GRADE approach. Evidence for the five key outcomes, namely, any red blood cell transfusion, rescue red blood cell transfusion, MACE, all-cause mortality, and SAEs, was rated as moderate, indicating that the estimates were reasonably stable but could still be affected by future research. Evidence for any intravenous iron use, mean monthly intravenous iron dose, and change in hemoglobin from baseline was rated as low, primarily because of substantial heterogeneity and uncertainty related to risk of bias. The complete GRADE evidence profile is provided in Supplementary Table S11.

## 4 Discussion

This systematic review and meta-analysis of RCTs indicates that HIF-PHIs improve anemia-related outcomes and reduce red blood cell transfusion requirements in patients with CKD-associated anemia, without evidence of increased cardiovascular risk over short- to medium-term follow-up.

The KDIGO 2026 Clinical Practice Guideline for the Management of Anemia in Chronic Kidney Disease considers HIF-PHIs alongside ESAs as treatment options (Babitt et al., 2026). Our analysis extends this framework by showing approximate relative reductions of 26% in any red blood cell transfusion and 30% in rescue transfusion, together with a trend toward reduced intravenous iron use. By quantifying reduced dependence on invasive supportive therapy, these findings provide a dimension of clinical net benefit beyond hemoglobin correction alone and may inform more specific treatment recommendations. HIF-PHIs are oral small molecules that inhibit prolyl hydroxylases and stabilize HIF-α, particularly HIF-1α and HIF-2α, thereby stimulating endogenous EPO production and pathways that increase intestinal iron absorption and mobilize stored iron (Li et al., 2025; Mennerich et al., 2026). The observed reductions in transfusion and intravenous iron requirements are consistent with this pharmacologic mechanism, which coordinates endogenous erythropoiesis and iron utilization more physiologically. In addition to achieving hemoglobin efficacy that is non-inferior to ESAs (Singh et al., 2021a), oral administration may reduce treatment burden. Taken together, HIF-PHIs offer a mechanistically novel and convenient strategy that may improve CKD anemia management by correcting anemia and reducing reliance on invasive supportive treatment.

Although the transfusion- and iron-sparing effects of HIF-PHIs are most directly explained by HIF stabilization, stimulation of endogenous erythropoietin production, and improved iron availability, additional mechanisms may contribute. CKD is characterized by interconnected oxidative, inflammatory, profibrotic, and metabolic disturbances. Recent mechanistic studies and reviews have implicated aryl hydrocarbon receptor-associated dysregulation of NF-κB and Nrf2 signaling in oxidative stress and inflammation, intrarenal renin–angiotensin system activation coupled to Wnt1/β-catenin signaling in podocyte injury, TGF-β/Smad3 signaling in renal inflammation and fibrosis, Smad3-mediated suppression of GPX4 and ferroptosis, and phosphatidylcholine metabolic disruption involving phospholipase A_2_ (Wang et al., 2023a; Miao et al., 2024; Hong et al., 2025; He et al., 2024; Liu K. et al., 2025; Wang et al., 2023b). These pathways may aggravate renal hypoxia, inflammation-associated iron restriction, erythropoietic hyporesponsiveness, and the loss or phenotypic transformation of renal erythropoietin-producing interstitial cells. By enhancing HIF-dependent adaptation to hypoxia, HIF-PHIs could theoretically improve redox and metabolic homeostasis and partially counter inflammation-associated iron restriction, thereby complementing their established effects on erythropoietin production and hepcidin-regulated iron availability (Li et al., 2025; Mennerich et al., 2026). However, the cited studies primarily define CKD pathobiology in disease-specific human samples and experimental models and do not demonstrate that HIF-PHIs directly inhibit the renin–angiotensin system/Wnt, TGF-β/Smad3, aryl hydrocarbon receptor/NF-κB/Nrf2, ferroptosis, or phospholipid-metabolism pathways. Therefore, the observed reductions in transfusion and intravenous iron requirements should be attributed primarily to enhanced endogenous erythropoiesis and iron utilization, whereas effects on these broader pathways remain biologically plausible hypotheses requiring direct mechanistic validation. Future studies should integrate serial measurements of erythropoietin, hepcidin, inflammatory and oxidative-stress markers, renin–angiotensin system and Wnt/β-catenin activity, TGF-β/Smad3–GPX4 signaling, and lipidomic profiles.

An exploratory difference in the iron-sparing effect was observed according to dialysis status. HIF-PHIs significantly reduced any intravenous iron use in NDD-CKD (RR = 0.54, 95% CI: 0.33–0.88; I^2^ = 0%), whereas the reduction was not statistically significant in DD-CKD (RR = 0.66, 95% CI: 0.31–1.43; I^2^ = 96.6%) (Barratt et al., 2021; Csiky et al., 2021; Nangaku et al., 2021b; Yamamoto et al., 2021a; Akizawa et al., 2021b). Several stage-dependent mechanisms may contribute to this pattern. In NDD-CKD, residual kidney function and the absence of recurrent dialysis-related blood loss may allow HIF-mediated reductions in hepcidin, enhanced intestinal iron absorption, increased transferrin availability, and mobilization of stored iron to translate more directly into lower exogenous iron requirements (Badura et al., 2024; Babitt and Lin, 2012; Li et al., 2025; Xie et al., 2024). In DD-CKD, more severe inflammation and uremia, markedly impaired renal hepcidin clearance, functional iron sequestration, recurrent procedural and extracorporeal blood loss, and greater reliance on protocolized intravenous iron may attenuate or obscure these effects. Intermittent dialytic removal of hepcidin and differences between dialysis modalities may further increase variability in iron availability. Nevertheless, this subgroup contrast should be regarded as hypothesis-generating rather than definitive evidence of effect modification. Only three NDD-CKD trials and two DD-CKD trials contributed to this outcome, and the DD-CKD estimate combined different HIF-PHIs with divergent drug-specific effects. Future prospective studies should use standardized iron-management protocols and serially assess hepcidin, erythroferrone, ferritin, transferrin saturation, transferrin, inflammatory markers, residual kidney function, and objectively measured blood loss across CKD stages and dialysis modalities.

The substantial heterogeneity in intravenous iron outcomes is likely to reflect not only differences in pharmacologic efficacy but also variation in trial-specific iron-management strategies. Across trials, intravenous iron could be initiated, withheld, or resumed according to different ferritin or transferrin saturation thresholds, while dosing ranged from fixed or protocol-driven schedules to rescue or investigator-directed administration. Differences in monitoring frequency, concomitant oral iron use, dialysis status, and local clinical practice may have further influenced cumulative iron exposure. These co-interventions may dilute or amplify between-group differences and thereby obscure the true iron-sparing effect of HIF-PHIs. Future trials should therefore adopt prespecified and standardized iron-management protocols, including harmonized ferritin and transferrin saturation thresholds, consistent dosing algorithms, uniform monitoring and assessment windows, and standardized reporting of cumulative intravenous iron exposure.

Previous studies have raised concerns that high ESA doses or high hemoglobin targets may increase cardiovascular risk, making direct evaluation of HIF-PHI cardiovascular safety in RCTs essential (Singh et al., 2021a; Locatelli et al., 2022). Cardiovascular safety is, therefore, a central consideration in the clinical use of HIF-PHIs. In the present meta-analysis, HIF-PHIs corrected anemia and reduced transfusion requirements without increasing the risks of MACE (RR = 1.01, 95% CI: 0.95–1.06) or all-cause mortality (RR = 1.05, 95% CI: 0.96–1.14). Although the overall risk of hyperkalemia was not significantly increased, a clinically relevant signal was observed in patients with NDD-CKD (RR = 1.39, 95% CI: 1.05–1.83; I^2^ = 0%) (Shutov et al., 2021; Coyne et al., 2021; Chen et al., 2019). This estimate was derived from only three placebo-controlled roxadustat trials, and all five trials reporting hyperkalemia evaluated roxadustat; therefore, the finding should be considered exploratory and cannot establish whether the increased risk is specific to roxadustat, represents a class effect of HIF-PHIs, or is influenced by between-trial differences in baseline potassium, kidney function, diabetes, concomitant renin–angiotensin system inhibitor use, or regional dietary potassium intake. Nevertheless, the signal warrants clinical attention because patients with NDD-CKD have impaired renal potassium excretion and often have additional risk factors for hyperkalemia. Before initiating an HIF-PHI, clinicians should assess serum potassium, bicarbonate, and kidney function and review any history of hyperkalemia and concomitant medications or supplements that may increase potassium. As a pragmatic, risk-adapted approach, serum potassium may be reassessed within 1–2 weeks in patients with high-normal baseline potassium, eGFR <30 mL/min/1.73 m^2^, previous hyperkalemia, diabetes, heart failure, metabolic acidosis, or concomitant use of renin–angiotensin–aldosterone system inhibitors, mineralocorticoid receptor antagonists, or potassium-sparing diuretics, and within 4 weeks in other patients with NDD-CKD. Potassium should also be reassessed after HIF-PHI dose escalation, changes in potassium-modifying medications, intercurrent illness, or deterioration in kidney function. If serum potassium increases, the result should be confirmed, reversible causes should be identified and corrected, and decisions regarding treatment continuation, temporary interruption, or dose modification should be individualized according to the severity of hyperkalemia and local management protocols. Because these monitoring intervals have not been prospectively validated, they should be regarded as pragmatic recommendations requiring further evaluation. Future trials and individual-patient-data analyses should prospectively examine hyperkalemia according to baseline potassium, kidney function, potassium-modifying medications, dietary factors, and event severity.

Importantly, the available safety evidence was not confined to the 6–12-month efficacy windows. Several roxadustat studies contributed randomized follow-up of 2–4 years, ASCEND-ND contributed a median of 1.9 years of adjudicated cardiovascular follow-up, and the DIALOGUE extension studies provided open-label molidustat exposure for up to 36 months (Akizawa et al., 2019; Fishbane et al., 2021; Shutov et al., 2021; Barratt et al., 2021; Csiky et al., 2021; Coyne et al., 2021; Singh et al., 2021b). These longer observations did not reveal a consistent new cardiovascular safety signal, but they were concentrated in a limited number of drug programs and were not designed to exclude rare or delayed harms. Malignancy warrants particular caution because the expected latency may exceed the available follow-up. In the ASCEND-ND *post hoc* analysis, follow-up through the end of the study yielded an HR of 1.04 (95% CI: 0.77–1.40) for cancer-related adverse events, whereas an analysis accounting for the different comparator dosing intervals yielded an HR of 1.12 (95% CI: 0.81–1.56). No excess risk was observed in ASCEND-D across the analytical approaches examined (Singh et al., 2023). These findings are reassuring, but not definitive, and longer comparative follow-up, cancer-specific surveillance, and post-marketing pharmacovigilance remain necessary.

These findings have direct clinical implications. Reducing red blood cell transfusion may lower the risks of transfusion-related infection, hemolytic reactions, and iron overload (Linder et al., 2021) while conserving blood resources; reducing intravenous iron exposure may also limit iron overload and oxidative stress (Xie et al., 2024); and the neutral pooled estimates for MACE and all-cause mortality provide relevant short- to medium-term safety evidence. HIF-PHIs, therefore, appear to offer anemia correction that is not inferior to ESAs, with the additional potential for reducing dependence on invasive supportive treatments, without increasing MACE or all-cause mortality during the available follow-up.

The generalizability of these findings to Western populations should be interpreted cautiously. A substantial proportion of the included trials were conducted exclusively in Asia, particularly in Japan and China, where hemoglobin treatment targets, background ESA exposure, iron-supplementation protocols, rescue-treatment criteria, and transfusion practices may differ from those used in Western healthcare systems. Conversely, several of the largest included trials were multinational and enrolled participants across geographically diverse settings, providing some support for broader applicability. We considered an exploratory subgroup analysis by recruitment region but did not perform a quantitative comparison because the available study-level data could not reliably isolate an independent regional effect. For the principal outcomes, few Asia-only studies were available, multinational trials did not report the relevant outcomes separately for Asian and Western participants, and recruitment region was closely associated with the HIF-PHI evaluated, dialysis status, comparator, and trial-specific anemia-management protocol. A regional subgroup analysis would therefore have been vulnerable to limited statistical power, ecological misclassification, and substantial confounding. Accordingly, the relative treatment effects may be informative across healthcare settings, but the absolute reductions in transfusion and intravenous iron use may vary according to local baseline event rates, hemoglobin targets, ESA use, iron-management practices, rescue-treatment criteria, and transfusion thresholds.

This study has several limitations. First, substantial heterogeneity was observed for any intravenous iron use and mean monthly intravenous iron dose. Because trial-level iron-management criteria, including ferritin or transferrin saturation thresholds and dosing algorithms, were incompletely reported and could not be systematically extracted, we were unable to formally investigate these potential sources of heterogeneity through subgroup analysis or meta-regression. The pooled iron-related estimates should, therefore, be interpreted cautiously. Second, although some roxadustat trials had 2–4 years of randomized follow-up and molidustat extensions provided up to 36 months of exposure, most assessments were limited to 24–52 weeks, and longer-term data were concentrated in a few drug programs and selected completers. Rare or delayed cardiovascular and malignancy risks therefore remain uncertain. Third, hyperkalemia data were available only from five roxadustat trials, while baseline potassium, renin-angiotensin system inhibitor use, and dietary potassium exposure were inconsistently reported, preventing drug-specific and effect-modifier analyses. Fourth, the predominance of single-country Asian studies, particularly from Japan and China, and the lack of region-specific participant data may limit generalizability to Western settings; a regional subgroup analysis was not reliable because region was confounded by drug, dialysis status, comparator, and local management protocols. Finally, because some active-controlled or open-label trials did not use matched placebos and differed in follow-up and treatment management, pharmacologic effects could not be fully separated from the additional attention associated with more intensive monitoring in the intervention groups.

## 5 Conclusion

As the global burden of CKD increases and limitations of conventional anemia therapies persist, effective, convenient oral treatments with acceptable overall safety remain needed. This systematic review and meta-analysis shows that HIF-PHIs are effective for correcting anemia in CKD. Beyond increasing hemoglobin, HIF-PHIs produced a clinically meaningful reduction in red blood cell transfusion, showed a trend toward reduced intravenous iron use, and did not increase short- to medium-term risks of MACE or all-cause mortality. However, the hyperkalemia signal observed in NDD-CKD warrants particular clinical attention. Serum potassium and kidney function should be assessed before treatment and reassessed early after HIF-PHI initiation and after dose or concomitant medication changes, with closer monitoring in patients with advanced CKD, high-normal baseline potassium, previous hyperkalemia, metabolic acidosis, diabetes, heart failure, or concomitant potassium-raising therapies. Treatment decisions should be individualized if hyperkalemia develops. Longer-term studies and post-marketing surveillance are needed to determine whether this signal represents a drug-specific or class-wide effect and to establish optimal monitoring intervals. Clinicians should consider the potential to reduce transfusion dependence when individualizing treatment. Future research should prioritize long-term cardiovascular outcome studies, dedicated malignancy surveillance, detailed analyses of clinically relevant subgroups, and pharmacoeconomic evaluations to define the role of HIF-PHIs across healthcare settings.

## Data Availability

The original contributions presented in the study are included in the article/Supplementary Material; further inquiries can be directed to the corresponding author.
