# Supplementary material for: Effects of hypoxia-inducible factor prolyl hydroxylase inhibitors on transfusion and intravenous iron use in chronic kidney disease anemia: a systematic review and meta-analysis
Source: Front Pharmacol. 2026 Sep 16;17:1916346. doi: 10.3389/fphar.2026.1916346 (PMC13623900; doi:10.3389/fphar.2026.1916346)
Supplement: Supplementary file 1 [file Supplementaryfile1.docx]

***Supplementary Material***

# Contents

eTable 1. Full electronic search strategy

eTable 2. Trial-to-comparison mapping and sample-size allocation

eTable 3. Quality assessment of included studies using the Cochrane Risk of Bias 2 tool

eTable 4. Primary meta-analysis pooled estimates

eTable 5. Subgroup analyses by dialysis status

eTable 6. Subgroup analyses by HIF-PHI

eTable 7. Supplementary pooled estimates for secondary and safety outcomes

eTable 8. Sensitivity analyses restricted to studies at low risk of bias

eTable 9. Summary of leave-one-out sensitivity analyses

eTable 10. Descriptive summary of continuous outcomes with substantial heterogeneity

eTable 11. GRADE evidence profile

eFigure 1. Forest plot of any red blood cell transfusion stratified by HIF-PHI

eFigure 2. Forest plot of rescue red blood cell transfusion stratified by HIF-PHI

eFigure 3. Forest plot of mean monthly intravenous iron dose

eFigure 4. Forest plot of any intravenous iron use stratified by HIF-PHI

eFigure 5. Forest plot of mean monthly intravenous iron dose stratified by HIF-PHI

eFigure 6. Forest plot of change in hemoglobin from baseline

eFigure 7. Forest plot of hemoglobin level during treatment

eFigure 8. Forest plot of change in ferritin from baseline

eFigure 9. Forest plot of any adverse event

eFigure 10. Forest plot of hyperkalemia

eFigure 11. Funnel plot for any red blood cell transfusion

eFigure 12. Funnel plot for major adverse cardiovascular events

eFigure 13. Funnel plot for any adverse event

eFigure 14. Funnel plot for all-cause mortality

eFigure 15. Funnel plot for serious adverse events

eTable 1. Full electronic search strategy

PubMed, Embase, and Web of Science were searched from database inception through April 10, 2026. Complete search strings and the number of records retrieved at each step are reported below.

## **PubMed**

| **No.** | **Search terms** | **Number of hits** |
| --- | --- | --- |
| #1 | (anemia[All Fields] OR anaemia[All Fields] OR haemoglobin[All Fields] OR hematocrit[All Fields] OR haematocrit[All Fields]) | 540,702 |
| #2 | ("kidney diseases"[MeSH:noexp] OR "Hypertension, Renal"[Mesh] OR "Kidney Cortex Necrosis"[Mesh] OR "Kidney Diseases, Cystic"[Mesh] OR "Kidney Papillary Necrosis"[Mesh] OR "Nephritis"[Mesh] OR "Nephrocalcinosis"[Mesh] OR "Nephrolithiasis"[Mesh] OR "Nephrosclerosis"[Mesh] OR "Nephrosis"[Mesh] OR "Perinephritis"[Mesh] OR "Renal Artery Obstruction"[Mesh] OR "Renal Insufficiency"[Mesh] OR "Uremia"[Mesh] OR "Renal Nutcracker Syndrome"[Mesh] OR "Renal Tubular Transport, Inborn Errors"[Mesh] OR "Zellweger Syndrome"[Mesh] OR "kidney disease"[All Fields] OR "kidney diseases"[All Fields] OR "renal disease"[All Fields] OR "renal diseases"[All Fields] OR "CKD"[All Fields] OR "kidney insufficiency"[All Fields] OR "kidney insufficiencies"[All Fields] OR "renal insufficiency"[All Fields] OR "renal insufficiencies"[All Fields] OR "kidney failure"[All Fields] OR "renal failure"[All Fields] OR "kidney injury"[All Fields] OR "kidney injuries"[All Fields] OR "renal injury"[All Fields] OR "renal injuries"[All Fields] OR "kidney dysfunction"[All Fields] OR "renal dysfunction"[All Fields] OR "glomerular dysfunction"[All Fields] OR "tubular dysfunction"[All Fields] OR "impaired kidney function"[All Fields] OR "impaired renal function"[All Fields] OR "renal impairment"[All Fields] OR "renal impairments"[All Fields] OR "kidney impairment"[All Fields] OR "nephropathy"[All Fields] OR "nephropathies"[All Fields] OR "renal hypertension"[All Fields] OR "renovascular hypertension"[All Fields] OR "renovascular disease"[All Fields] OR "kidney cortex necrosis"[All Fields] OR "renal cortex necrosis"[All Fields] OR "cystic kidney"[All Fields] OR "cystic kidneys"[All Fields] OR "kidney cyst"[All Fields] OR "kidney cysts"[All Fields] OR "renal cyst"[All Fields] OR "renal cysts"[All Fields] OR "multicystic kidney"[All Fields] OR "polycystic kidney"[All Fields] OR "sponge kidney"[All Fields] OR "ADPKD"[All Fields] OR "ARPKD"[All Fields] OR "PKD"[All Fields] OR "dysplastic kidney"[All Fields] OR "kidney dysplasia"[All Fields] OR "kidney dysplasias"[All Fields] OR "renal dysplasia"[All Fields] OR "renal dysplasias"[All Fields] OR "hypoplastic kidney"[All Fields] OR "kidney hypoplasia"[All Fields] OR "kidney hypoplasias"[All Fields] OR "renal hypoplasia"[All Fields] OR "renal hypoplasias"[All Fields] OR "kidney hypertroph"[All Fields] OR "renal hypertrophy"[All Fields] OR "kidney malformation"[All Fields] OR "renal malformation"[All Fields] OR "kidney papillary necrosis"[All Fields] OR "renal necrosis"[All Fields] OR "kidney papilla necrosis"[All Fields] OR "nephritis"[All Fields] OR "glomerulonephritis"[All Fields] OR "glomerulitis"[All Fields] OR "pyelonephritis"[All Fields] OR "pyelitis"[All Fields] OR "nephrocalcinosis"[All Fields] OR "nephrolithiasis"[All Fields] OR "nephrosclerosis"[All Fields] OR "nephrosis"[All Fields] OR "nephrotic"[All Fields] OR "perinephritis"[All Fields] OR "perinephritic"[All Fields] OR "renal artery obstruction"[All Fields] OR "acute kidney injury"[All Fields] OR "AKI"[All Fields] OR "renal nutcracker syndrome"[All Fields] OR "tubular acidosis"[All Fields] OR "tubule acidosis"[All Fields] OR "tubule damage"[All Fields] OR "tubule disorder"[All Fields] OR "Bartter"[All Fields] OR "Dent Disease"[All Fields] OR "Fanconi syndrome"[All Fields] OR "Gitelman syndrome"[All Fields] OR "renal glycosuria"[All Fields] OR "hypophosphatemia"[All Fields] OR "Liddle syndrome"[All Fields] OR "oculocerebrorenal syndrome"[All Fields] OR "pseudohypoaldosteronism"[All Fields] OR "renal aminoacidurias"[All Fields] OR "azotemia"[All Fields] OR "hemolytic-uremic syndrome"[All Fields] OR "aHUS"[All Fields] OR "HUS"[All Fields] OR "uremia"[All Fields] OR "Zellweger syndrome"[All Fields] OR "tubular necrosis"[All Fields] OR "Alport"[All Fields] OR "COL4A5"[All Fields] OR "COL4A4"[All Fields] OR "COL4A3"[All Fields] OR "Fabry"[All Fields] OR "FSGS"[All Fields] OR "focal segmental glomerulosclerosis"[All Fields] OR "Henoch-Schönlein"[All Fields] OR "Berger"[All Fields] OR "Wegener"[All Fields] OR "granulomatosis"[All Fields] OR "microangiopathy"[All Fields] OR "microangiopathies"[All Fields] OR "TMA"[All Fields] OR "nephronophthisis"[All Fields] OR "nephroptosis"[All Fields] OR "cystinuria"[All Fields] OR "cysteine stone"[All Fields] OR "renal fibrosis"[All Fields] OR "kidney fibrosis"[All Fields] OR "kidney amyloidosis"[All Fields] OR "renal amyloidosis"[All Fields] OR "kidney calcification"[All Fields] OR "renal calcification"[All Fields] OR "kidney ischem"[All Fields] OR "renal ischem"[All Fields] OR "kidney necrosis"[All Fields] OR "kidney rupture"[All Fields] OR "ciliopathy*"[All Fields]) OR ("chronic kidney disease"[All Fields] OR "chronic renal disease"[All Fields] OR "end stage kidney disease"[All Fields] OR "end stage renal disease"[All Fields] OR "end stage renal failure"[All Fields] OR "ESRF"[All Fields]) | 900,830 |
| #3 | (("Hypoxia-Inducible Factor-Prolyl Hydroxylase Inhibitors"[Mesh]) OR ("HIF-prolyl hydroxylase inhibitor"[All Fields] OR "prolyl hydroxylase inhibitor"[All Fields] OR "HIF-PH inhibitor*"[All Fields] OR "HIF-PHDi"[All Fields]) OR ("PHD inhibitor"[All Fields] OR "hypoxia inducible factor stabilizer"[All Fields] OR "HIF stabilizer"[All Fields] OR "HIF-PHIs"[All Fields])) OR (("Roxadustat"[All Fields] OR "FG-4592"[All Fields] OR "ASP1517"[All Fields]) OR ("Vadadustat"[All Fields] OR "AKB-6548"[All Fields]) OR ("Daprodustat"[All Fields] OR "GSK1278863"[All Fields]) OR ("Molidustat"[All Fields] OR "BAY 85-3934"[All Fields]) OR ("Enarodustat"[All Fields] OR "JTZ-951"[All Fields]) OR ("Desidustat"[All Fields] OR "ZYAN1"[All Fields]) OR ("JNJ-42905343"[All Fields]) OR ("TP0463518"[All Fields]) OR ("AKB-4924"[All Fields]) OR ("DDO-3055"[All Fields]) OR ("MK-8617"[All Fields]) OR ("BGE-117"[All Fields])) | 1,358 |
| #4 | ("randomized controlled trial"[pt] OR "controlled clinical trial"[pt] OR randomized[tiab] OR randomised[tiab] OR placebo[tiab] OR "drug therapy"[sh] OR randomly[tiab] OR trial[tiab] OR groups[tiab]) NOT ("animals"[mh] NOT "humans"[mh]) | 5,930,929 |
| #5 | #1 AND #2 AND #3 AND #4 Filter: Humans | 428 |

## **Embase**

| **No.** | **Search terms** | **Number of hits** |
| --- | --- | --- |
| #1 | 'anemia'/exp OR 'hemoglobin'/exp OR ('anaemia':ab,ti OR 'anemia':ab,ti OR 'anaemia gravis':ab,ti OR 'anemia gravis':ab,ti OR 'chronic anaemia':ab,ti OR 'chronic anemia':ab,ti OR 'normoblastic anaemia':ab,ti OR 'normoblastic anemia':ab,ti OR 'secondary anaemia':ab,ti OR 'secondary anemia':ab,ti) | 1,122,550 |
| #2 | ('kidney disease'/de OR 'cystinuria'/exp OR 'experimental kidney disease'/exp OR 'Fabry disease'/exp OR 'Gitelman syndrome'/exp OR 'glomerulopathy'/exp OR 'Gordon syndrome'/exp OR 'hypertensive nephropathy'/exp OR 'kidney amyloidosis'/exp OR 'kidney calcification'/exp OR 'kidney cyst'/exp OR 'kidney dysfunction'/exp OR 'kidney failure'/exp OR 'kidney fibrosis'/exp OR 'kidney hemorrhage'/exp OR 'kidney hypertrophy'/exp OR 'kidney infarction'/exp OR 'kidney infection'/exp OR 'kidney injury'/exp OR 'kidney ischemia'/exp OR 'kidney malformation'/exp OR 'kidney necrosis'/exp OR 'kidney papilla necrosis'/exp OR 'kidney polycystic disease'/exp OR 'kidney rupture'/exp OR 'kidney scar'/exp OR 'kidney tubule acidosis'/exp OR 'kidney tubule damage'/exp OR 'kidney tubule disorder'/exp OR 'Liddle syndrome'/exp OR 'Lowe syndrome'/exp OR 'Meckel syndrome'/exp OR 'medullary sponge kidney'/exp OR 'nephritis'/exp OR 'nephrolithiasis'/exp OR 'nephrosis'/exp OR 'nephrotoxicity'/exp OR 'reflux nephropathy'/exp OR 'renal graft dysfunction'/exp OR 'renovascular disease'/exp OR 'uric acid nephropathy'/exp OR 'renovascular hypertension'/exp OR 'kidney cortex necrosis'/exp OR 'ciliopathy'/exp OR 'kidney artery stenosis'/exp) OR ('kidney disease':ab,ti OR 'kidney diseases':ab,ti OR 'renal disease':ab,ti OR 'renal diseases':ab,ti OR 'CKD':ab,ti OR 'kidney insufficiency':ab,ti OR 'kidney insufficiencies':ab,ti OR 'renal insufficiency':ab,ti OR 'renal insufficiencies':ab,ti OR 'kidney failure':ab,ti OR 'renal failure':ab,ti OR 'kidney injury':ab,ti OR 'kidney injuries':ab,ti OR 'renal injury':ab,ti OR 'renal injuries':ab,ti OR 'kidney dysfunction':ab,ti OR 'renal dysfunction':ab,ti OR 'glomerular dysfunction':ab,ti OR 'tubular dysfunction':ab,ti OR 'impaired kidney function':ab,ti OR 'impaired renal function':ab,ti OR 'renal impairment':ab,ti OR 'renal impairments':ab,ti OR 'kidney impairment':ab,ti OR 'nephropathy':ab,ti OR 'nephropathies':ab,ti OR 'renal hypertension':ab,ti OR 'renovascular hypertension':ab,ti OR 'renovascular disease':ab,ti OR 'kidney cortex necrosis':ab,ti OR 'renal cortex necrosis':ab,ti OR 'cystic kidney':ab,ti OR 'cystic kidneys':ab,ti OR 'kidney cyst':ab,ti OR 'kidney cysts':ab,ti OR 'renal cyst':ab,ti OR 'renal cysts':ab,ti OR 'multicystic kidney':ab,ti OR 'polycystic kidney':ab,ti OR 'sponge kidney':ab,ti OR 'ADPKD':ab,ti OR 'ARPKD':ab,ti OR 'PKD':ab,ti OR 'dysplastic kidney':ab,ti OR 'kidney dysplasia':ab,ti OR 'kidney dysplasias':ab,ti OR 'renal dysplasia':ab,ti OR 'renal dysplasias':ab,ti OR 'hypoplastic kidney':ab,ti OR 'kidney hypoplasia':ab,ti OR 'kidney hypoplasias':ab,ti OR 'renal hypoplasia':ab,ti OR 'renal hypoplasias':ab,ti OR 'kidney hypertroph':ab,ti OR 'renal hypertroph':ab,ti OR 'kidney malformation':ab,ti OR 'renal malformation':ab,ti OR 'kidney papillary necrosis':ab,ti OR 'renal necrosis':ab,ti OR 'kidney papilla necrosis':ab,ti OR 'renal papilla necrosis':ab,ti OR 'nephritis':ab,ti OR 'glomerulonephritis':ab,ti OR 'glomerulitis':ab,ti OR 'pyelonephritis':ab,ti OR 'pyelitis':ab,ti OR 'nephrocalcinosis':ab,ti OR 'nephrolithiasis':ab,ti OR 'nephrosclerosis':ab,ti OR 'nephrosis':ab,ti OR 'nephrotic':ab,ti OR 'perinephritis':ab,ti OR 'perinephritic':ab,ti OR 'renal artery obstruction':ab,ti OR 'acute kidney injury':ab,ti OR 'AKI':ab,ti OR 'renal nutcracker syndrome':ab,ti OR 'tubular acidosis':ab,ti OR 'tubule acidosis':ab,ti OR 'tubule damage':ab,ti OR 'tubule disorder':ab,ti OR 'Bartter':ab,ti OR 'Dent disease':ab,ti OR 'Fanconi syndrome':ab,ti OR 'Gitelman syndrome':ab,ti OR 'renal glycosuria':ab,ti OR 'hypophosphatemia':ab,ti OR 'Liddle syndrome':ab,ti OR 'oculocerebrorenal syndrome':ab,ti OR 'pseudohypoaldosteronism':ab,ti OR 'renal aminoacidurias':ab,ti OR 'azotemia':ab,ti OR 'hemolytic-uremic syndrome':ab,ti OR 'aHUS':ab,ti OR 'HUS':ab,ti OR 'uremia':ab,ti OR 'Zellweger syndrome':ab,ti OR 'tubular necrosis':ab,ti OR 'Alport':ab,ti OR 'COL4A5':ab,ti OR 'COL4A4':ab,ti OR 'COL4A3':ab,ti OR 'Fabry':ab,ti OR 'FSGS':ab,ti OR 'focal segmental glomerulosclerosis':ab,ti OR 'Henoch-Schonlein':ab,ti OR 'Berger':ab,ti OR 'Wegener':ab,ti OR 'granulomatosis':ab,ti OR 'microangiopathy':ab,ti OR 'microangiopathies':ab,ti OR 'TMA':ab,ti OR 'nephronophthisis':ab,ti OR 'nephroptosis':ab,ti OR 'cystinuria':ab,ti OR 'cysteine stone':ab,ti OR 'renal fibrosis':ab,ti OR 'kidney fibrosis':ab,ti OR 'kidney amyloidosis':ab,ti OR 'renal amyloidosis':ab,ti OR 'kidney calcification':ab,ti OR 'renal calcification':ab,ti OR 'kidney ischem':ab,ti OR 'renal ischem':ab,ti OR 'kidney necrosis':ab,ti OR 'kidney rupture':ab,ti OR 'ciliopath*':ab,ti) | 1,524,754 |
| #3 | ('prolyl hydroxylase inhibitor'/exp OR 'hypoxia inducible factor 1'/exp) OR ('HIF-PHI':ab,ti OR 'HIF PHI':ab,ti OR 'hypoxia-inducible factor prolyl hydroxylase inhibitor':ab,ti OR 'hypoxia-inducible factor prolyl hydroxylase inhibitors':ab,ti OR 'Hypoxia Inducible Factor 1 alpha Subunit':ab,ti OR 'prolyl hydroxylase inhibitor':ab,ti OR 'prolyl hydroxylase inhibitors':ab,ti OR 'proline dioxygenase inhibitor':ab,ti OR 'proline dioxygenase inhibitors':ab,ti OR 'hypoxia inducible factor stabilizer':ab,ti OR 'HIF stabilizer':ab,ti OR 'HIF stabiliser':ab,ti OR 'HIF-PH inhibitor*':ab,ti OR 'HIF-PHDi':ab,ti OR 'PHD inhibitor':ab,ti) OR ('Roxadustat':ab,ti OR 'FG-4592':ab,ti OR 'ASP1517':ab,ti OR 'Vadadustat':ab,ti OR 'AKB-6548':ab,ti OR 'Daprodustat':ab,ti OR 'GSK1278863':ab,ti OR 'Molidustat':ab,ti OR 'BAY 85-3934':ab,ti OR 'Enarodustat':ab,ti OR 'JTZ-951':ab,ti OR 'Desidustat':ab,ti OR 'ZYAN1':ab,ti OR 'JNJ-42905343':ab,ti OR 'TP0463518':ab,ti OR 'AKB-4924':ab,ti OR 'DDO-3055':ab,ti OR 'MK-8617':ab,ti OR 'BGE-117':ab,ti OR 'Duvroq':ab,ti OR 'evrenzo':ab,ti) | 16,175 |
| #4 | 'randomized controlled trial'/exp OR 'controlled clinical study'/exp OR (random* NEAR/3 (allocat* OR assign* OR control* OR group* OR trial)):ab,ti OR (placebo:ab,ti AND (trial:ab,ti OR study:ab,ti)) OR ((single OR double OR triple OR treble) NEAR/3 (blind* OR mask*)):ab,ti OR (controlled NEAR/3 (study OR design OR trial)):ab,ti OR (cross over:ab,ti OR crossover:ab,ti) OR (volunteer:ab,ti OR volunteers:ab,ti) OR (trial:ti OR random*:ti) OR (comparative NEAR/3 study):ab,ti | 3,002,636 |
| #5 | #1 AND #2 AND #3 AND #4 | 516 |

## **Web of Science**

| **No.** | **Search terms** | **Number of hits** |
| --- | --- | --- |
| #1 | TS=(anemia OR anaemia OR haemoglobin OR hematocrit OR haematocrit) | 862,799 |
| #2 | TS=("kidney disease*" OR "renal disease*" OR CKD OR "kidney insufficienc*" OR "renal insufficienc*" OR "kidney failure" OR "renal failure" OR "kidney injur*" OR "renal injur*" OR "kidney dysfunction" OR "renal dysfunction" OR "glomerular dysfunction" OR "tubular dysfunction" OR "impaired kidney function" OR "impaired renal function" OR "renal impairment*" OR "kidney impairment*" OR nephropath* OR "renal hypertension" OR "renovascular hypertension" OR "renovascular disease" OR "kidney cortex necrosis" OR "renal cortex necrosis" OR "cystic kidney" OR "kidney cyst*" OR "renal cyst*" OR "multicystic kidney*" OR "polycystic kidney*" OR "sponge kidney*" OR ADPKD OR ARPKD OR PKD OR "dysplastic kidney*" OR "kidney dysplasia" OR "renal dysplasia" OR "hypoplastic kidney*" OR "kidney hypoplasia" OR "renal hypoplasia" OR "kidney hypertroph*" OR "renal hypertroph*" OR "kidney malformation*" OR "renal malformation*" OR "kidney papillary necrosis" OR "renal necrosis" OR "kidney papilla necrosis" OR nephritis OR glomerulonephritis OR glomerulitis OR pyelonephritis OR pyelitis OR nephrocalcinosis OR nephrolithiasis OR nephrosclerosis OR nephrosis OR nephrotic OR perinephritis OR perinephritic OR "renal artery obstruction" OR "acute kidney injury" OR AKI OR "renal nutcracker syndrome" OR "tubular acidosis" OR "tubule acidosis" OR "tubule damage" OR "tubule disorder" OR Bartter OR "Dent Disease" OR "Fanconi syndrome" OR "Gitelman syndrome" OR "renal glycosuria" OR hypophosphatemia OR "Liddle syndrome" OR "oculocerebrorenal syndrome" OR pseudohypoaldosteronism OR "renal aminoacidurias" OR azotemia OR "hemolytic-uremic syndrome" OR aHUS OR HUS OR uremia OR "Zellweger syndrome" OR "tubular necrosis" OR Alport OR COL4A5 OR COL4A4 OR COL4A3 OR Fabry OR FSGS OR "focal segmental glomerulosclerosis" OR "Henoch-Schönlein" OR Berger OR Wegener OR granulomatosis OR microangiopath* OR TMA OR nephronophthisis OR nephroptosis OR cystinuria OR "cysteine stone" OR "renal fibrosis" OR "kidney fibrosis" OR "kidney amyloidosis" OR "renal amyloidosis" OR "kidney calcification" OR "renal calcification" OR "kidney ischem*" OR "renal ischem*" OR "kidney necrosis" OR "kidney rupture" OR ciliopath* OR "chronic kidney disease" OR "chronic renal disease" OR "end stage kidney disease" OR "end stage renal disease" OR "end stage renal failure" OR ESRF) | 1,389,569 |
| #3 | TS=("Hypoxia-Inducible Factor-Prolyl Hydroxylase Inhibitor*" OR "HIF-prolyl hydroxylase inhibitor*" OR "prolyl hydroxylase inhibitor*" OR "HIF-PH inhibitor*" OR "HIF-PHDi" OR "PHD inhibitor" OR "hypoxia inducible factor stabilizer" OR "HIF stabilizer" OR "HIF-PHIs" OR Roxadustat OR "FG-4592" OR ASP1517 OR Vadadustat OR "AKB-6548" OR Daprodustat OR GSK1278863 OR Molidustat OR "BAY 85-3934" OR Enarodustat OR "JTZ-951" OR Desidustat OR ZYAN1 OR JNJ-42905343 OR TP0463518 OR "AKB-4924" OR "DDO-3055" OR "MK-8617" OR BGE-117) | 3,140 |
| #4 | TS=((random* NEAR/2 (allocat* OR assign* OR control* OR group* OR trial)) OR (placebo AND (trial OR study)) OR ((single OR double OR triple OR treble) NEAR/2 (blind* OR mask*)) OR (controlled NEAR/2 (study OR design OR trial)) OR (cross over OR crossover) OR (volunteer OR volunteers) OR (comparative NEAR/2 study)) OR TI=(trial OR random*) | 7,620,768 |
| #5 | #1 AND #2 AND #3 AND #4 | 439 |

eTable 2. Trial-to-comparison mapping and sample-size allocation

| **Study** | **Trial registration / comparison** | **Population** | **HIF-PHI** | **Comparator** | **N HIF-PHI** | **N control** |
| --- | --- | --- | --- | --- | --- | --- |
| Fishbane et al, 2022 | NCT02174731 | Incident + prevalent DD-CKD | Roxadustat | Epoetin alfa | 1068 | 1065 |
| Akizawa et al, 2019 | NCT01964196 | NDD-CKD | Roxadustat | Placebo | 80 | 27 |
| Hou et al, 2022 | ChiCTR2000035054 | PD-dependent CKD | Roxadustat | ESAs | 86 | 43 |
| Fishbane et al, 2021 | NCT02174627 | NDD-CKD | Roxadustat | Placebo | 1393 | 1388 |
| Shutov et al, 2021 | NCT01887600 | NDD-CKD | Roxadustat | Placebo | 391 | 203 |
| Barratt et al, 2021 | NCT02021318 | NDD-CKD | Roxadustat | Darbepoetin alfa | 323 | 293 |
| Csiky et al, 2021 | NCT02964936 | Prevalent DD-CKD | Roxadustat | ESA (epoetin alfa or darbepoetin alfa) | 415 | 421 |
| Coyne et al, 2021 | NCT01750190 | NDD-CKD | Roxadustat | Placebo | 616 | 306 |
| Chen et al, 2019 | NCT02652819 | NDD-CKD | Roxadustat | Placebo | 101 | 51 |
| Provenzano et al, 2021 | NCT02052310 | Incident DD-CKD (2 weeks to <=4 months) | Roxadustat | Epoetin alfa | 522 | 521 |
| Nangaku et al, 2021 | NCT03054337 | NDD-CKD | vadadustat | placebo | 37 | 14 |
| Nangaku et al, 2021 | NCT03054350 | DD-CKD | vadadustat | placebo | 44 | 14 |
| Pergola et al, 2016 | NCT01906489 | NDD-CKD | vadadustat | placebo | 138 | 72 |
| Toka et al, 2025 | NCT04707768 | DD-CKD | vadadustat | methoxy polyethylene glycol-epoetin-beta (MPG-EPO) | 304 | 152 |
| Chertow et al, 2021 | NCT02648347 | NDD-CKD, ESA-untreated | vadadustat | darbepoetin alfa | 879 | 872 |
| Chertow et al, 2021 | NCT02680574 | NDD-CKD, ESA-treated | vadadustat | darbepoetin alfa | 862 | 863 |
| Chertow et al, 2021 | NCT02648347 + NCT02680574 | NDD-CKD, pooled safety population | vadadustat | darbepoetin alfa | 1739 | 1732 |
| Coyne et al, 2022 | NCT03400033 | Prevalent HD-dependent CKD | daprodustat | epoetin alfa | 270 | 137 |
| Johansen et al, 2023 | NCT03409107 | NDD-CKD, stages 3-5 | daprodustat | placebo | 307 | 307 |
| Kooienga et al, 2024 | NCT04313153 | DD-CKD, converted from ESA | vadadustat | darbepoetin alfa | 105 | 108 |
| Kooienga et al, 2024 | NCT04313153 | DD-CKD, converted from ESA | vadadustat | darbepoetin alfa | 106 | 108 |
| Eckardt et al, 2021 | NCT02865850 | Incident DD-CKD | vadadustat | darbepoetin alfa | 181 | 188 |
| Eckardt et al, 2021 | NCT02892149 | Prevalent DD-CKD | vadadustat | darbepoetin alfa | 1777 | 1777 |
| Eckardt et al, 2021 | NCT02865850 + NCT02892149 | DD-CKD, pooled incident + prevalent safety population | vadadustat | darbepoetin alfa | 1947 | 1955 |
| Provenzano et al, 2016 | NCT01147666 | HD-dependent CKD | roxadustat | epoetin alfa | 67 | 23 |
| Agrawal et al, 2022 |  | NDD-CKD | desidustat | darbepoetin alfa | 294 | 294 |
| Gang et al, 2022 | CTRI/2019/12/022312 | DD-CKD | desidustat | epoetin alfa | 196 | 196 |
| Singh et al, 2021 | NCT02876835 | NDD-CKD | daprodustat | darbepoetin alfa | 1937 | 1935 |
| Holdstock et al, 2019 | NCT01977573 | NDD-CKD | daprodustat | rhEPO standard of care | 156 | 79 |
| Meadowcroft et al, 2019 | NCT01977482 | HD-dependent CKD | daprodustat | placebo for 4 weeks then open-label rhEPO as required | 171 | 39 |
| Nangaku et al, 2021 | NCT02791763 | NDD-CKD | daprodustat | epoetin beta pegol (CERA) | 108 | 109 |
| Charytan et al, 2021 | NCT02273726 | DD-CKD | roxadustat | epoetin alfa | 370 | 371 |
| Akizawa et al, 2021 | JapicCTI-183938 | HD-dependent CKD | enarodustat | darbepoetin alfa | 86 | 86 |
| Nangaku et al, 2021 | NCT03329196 | NDD-CKD | vadadustat | darbepoetin alfa | 151 | 153 |
| Chen et al, 2017 | NCT01599507 | NDD-CKD | roxadustat (FG-4592) | placebo | 61 | 30 |
| Chen et al, 2017 | NCT01596855 | DD-CKD | roxadustat (FG-4592) | epoetin alfa | 74 | 22 |
| Yamamoto et al, 2021 | NCT03350347 | NDD-CKD | molidustat | darbepoetin alfa | 82 | 82 |
| Akizawa et al, 2021 | NCT03543657 | DD-CKD | molidustat | darbepoetin alfa | 153 | 76 |
| Liang et al, 2024 |  | NDD-CKD | enarodustat | placebo | 103 | 52 |
| Yamamoto et al, 2021 | NCT03350321 | NDD-CKD | molidustat | darbepoetin alfa | 82 | 80 |
| Dasgupta et al, 2025 | NCT02879305 | PD-dependent CKD | daprodustat | darbepoetin alfa | 171 | 169 |
| Singh et al, 2022 | NCT03029208 | Incident DD-CKD, HD/PD | daprodustat | darbepoetin alfa | 157 | 155 |
| Akizawa et al, 2020 | NCT02969655 | DD-CKD | daprodustat | darbepoetin alfa | 136 | 135 |
| Macdougall et al, 2019 | NCT02021370 | NDD-CKD | molidustat | placebo | 101 | 20 |
| Macdougall et al, 2019 | NCT02021409 | NDD-CKD | molidustat | darbepoetin alfa | 92 | 32 |
| Macdougall et al, 2019 | NCT01975818 | DD-CKD | molidustat | epoetin alfa/beta | 157 | 42 |

eTable 3. Quality assessment of included studies using the Cochrane Risk of Bias 2 tool

eTable 4. Primary meta-analysis pooled estimates

| **Outcome** | **Effect** | **Number of studies (k)** | **Random-effects model (95% CI)** | **Fixed-effects model (95% CI)** | **Heterogeneity (I², %)** | **Cochran's Q p-value** |
| --- | --- | --- | --- | --- | --- | --- |
| Any intravenous iron use | RR | 5 | 0.66 (95% CI 0.38 to 1.14) | 0.63 (95% CI 0.55 to 0.72) | 87.3 | <0.001 |
| Any red blood cell transfusion | RR | 12 | 0.74 (95% CI 0.58 to 0.93) | 0.72 (95% CI 0.63 to 0.83) | 54.0 | 0.013 |
| Monthly intravenous iron dose | SMD | 5 | -0.19 (95% CI -0.42 to 0.03) | -0.19 (95% CI -0.26 to -0.13) | 89.2 | <0.001 |
| Rescue red blood cell transfusion | RR | 9 | 0.70 (95% CI 0.53 to 0.92) | 0.64 (95% CI 0.57 to 0.73) | 53.1 | 0.029 |

eTable 5. Subgroup analyses by dialysis status

| **Outcome** | **Dialysis status** | **Effect** | **Number of studies (k)** | **Random-effects model (95% CI)** | **Heterogeneity (I², %)** | **Cochran's Q p-value** |
| --- | --- | --- | --- | --- | --- | --- |
| Any intravenous iron use | Dialysis | RR | 2 | 0.66 (95% CI 0.31 to 1.43) | 96.6 | <0.001 |
| Any intravenous iron use | Non-dialysis | RR | 3 | 0.54 (95% CI 0.33 to 0.88) | 0.0 | 0.441 |
| Any red blood cell transfusion | Dialysis | RR | 7 | 0.74 (95% CI 0.64 to 0.86) | 0.0 | 0.482 |
| Any red blood cell transfusion | Non-dialysis | RR | 5 | 0.72 (95% CI 0.29 to 1.83) | 77.4 | 0.001 |
| Monthly intravenous iron dose | Dialysis | SMD | 3 | -0.13 (95% CI -0.45 to 0.18) | 92.4 | <0.001 |
| Monthly intravenous iron dose | Non-dialysis | SMD | 2 | -0.31 (95% CI -0.88 to 0.25) | 90.4 | 0.001 |
| Rescue red blood cell transfusion | Dialysis | RR | 2 | 0.74 (95% CI 0.60 to 0.90) | 0.0 | 0.866 |
| Rescue red blood cell transfusion | Non-dialysis + dialysis | RR | 2 | 0.12 (95% CI 0.02 to 0.74) | 0.0 | 0.818 |
| Rescue red blood cell transfusion | Non-dialysis | RR | 5 | 0.73 (95% CI 0.39 to 1.38) | 64.5 | 0.024 |
| Change in hemoglobin from baseline | Dialysis | MD | 4 | 0.20 (95% CI 0.09 to 0.30) | 49.7 | 0.113 |
| Change in hemoglobin from baseline | Non-dialysis | MD | 5 | 1.65 (95% CI 1.30 to 2.01) | 88.9 | <0.001 |
| Hemoglobin level during treatment | Dialysis | MD | 3 | -0.27 (95% CI -0.56 to 0.02) | 79.7 | 0.007 |
| MACE / cardiovascular events | Dialysis | RR | 10 | 0.96 (95% CI 0.89 to 1.04) | 0.0 | 0.746 |
| MACE / cardiovascular events | Non-dialysis | RR | 7 | 1.05 (95% CI 0.97 to 1.13) | 0.0 | 0.698 |
| Any adverse events | Dialysis | RR | 9 | 1.01 (95% CI 0.98 to 1.04) | 0.0 | 0.942 |
| Any adverse events | Non-dialysis | RR | 9 | 1.02 (95% CI 1.00 to 1.04) | 0.0 | 0.591 |
| Hyperkalemia | Dialysis | RR | 2 | 0.93 (95% CI 0.39 to 2.22) | 38.3 | 0.203 |
| Hyperkalemia | Non-dialysis | RR | 3 | 1.39 (95% CI 1.05 to 1.83) | 0.0 | 0.767 |
| Change in ferritin from baseline | Dialysis | SMD | 3 | -0.17 (95% CI -0.41 to 0.07) | 0.0 | 0.401 |
| Change in ferritin from baseline | Non-dialysis | SMD | 4 | -0.14 (95% CI -0.33 to 0.05) | 62.4 | 0.046 |
| Monthly intravenous iron dose | Dialysis | SMD | 4 | -0.13 (95% CI -0.29 to 0.04) | 69.7 | 0.019 |
| All-cause mortality | Dialysis | RR | 9 | 1.01 (95% CI 0.87 to 1.18) | 0.0 | 0.438 |
| All-cause mortality | Non-dialysis | RR | 9 | 1.06 (95% CI 0.96 to 1.18) | 0.0 | 0.669 |
| Serious adverse events | Dialysis | RR | 11 | 0.98 (95% CI 0.94 to 1.02) | 0.0 | 0.538 |
| Serious adverse events | Non-dialysis | RR | 8 | 1.10 (95% CI 1.04 to 1.17) | 24.4 | 0.234 |

eTable 6. Subgroup analyses by HIF-PHI

| **Outcome** | **Drug** | **Effect** | **Number of studies (k)** | **Random-effects model (95% CI)** | **Heterogeneity (I², %)** | **Cochran's Q p-value** |
| --- | --- | --- | --- | --- | --- | --- |
| Any intravenous iron use | Molidustat | RR | 2 | 0.98 (95% CI 0.80 to 1.21) | 0.0 | 0.986 |
| Any intravenous iron use | Roxadustat | RR | 2 | 0.45 (95% CI 0.38 to 0.54) | 0.0 | 0.761 |
| Any red blood cell transfusion | Daprodustat | RR | 4 | 0.80 (95% CI 0.54 to 1.20) | 0.0 | 0.547 |
| Any red blood cell transfusion | Molidustat | RR | 2 | 0.35 (95% CI 0.05 to 2.25) | 0.0 | 0.650 |
| Any red blood cell transfusion | Roxadustat | RR | 6 | 0.73 (95% CI 0.54 to 0.99) | 75.9 | <0.001 |
| Monthly intravenous iron dose | Molidustat | SMD | 2 | -0.17 (95% CI -1.04 to 0.70) | 94.2 | <0.001 |
| Monthly intravenous iron dose | Roxadustat | SMD | 3 | -0.21 (95% CI -0.44 to 0.01) | 89.6 | <0.001 |
| Rescue red blood cell transfusion | Molidustat | RR | 2 | 0.62 (95% CI 0.08 to 4.95) | 0.0 | 0.617 |
| Rescue red blood cell transfusion | Roxadustat | RR | 5 | 0.73 (95% CI 0.54 to 0.97) | 70.5 | 0.009 |
| Rescue red blood cell transfusion | Vadadustat | RR | 2 | 0.13 (95% CI 0.02 to 0.76) | 0.0 | 0.794 |
| Change in hemoglobin from baseline | Enarodustat | MD | 3 | 0.87 (95% CI -0.12 to 1.87) | 97.6 | <0.001 |
| Change in hemoglobin from baseline | Roxadustat | MD | 6 | 1.05 (95% CI 0.35 to 1.74) | 99.2 | <0.001 |
| Hemoglobin level during treatment | Vadadustat | MD | 2 | -0.40 (95% CI -0.67 to -0.12) | 53.8 | 0.141 |
| MACE / cardiovascular events | Daprodustat | RR | 6 | 1.03 (95% CI 0.91 to 1.16) | 0.0 | 0.776 |
| MACE / cardiovascular events | Roxadustat | RR | 5 | 0.97 (95% CI 0.87 to 1.07) | 4.6 | 0.381 |
| MACE / cardiovascular events | Vadadustat | RR | 5 | 1.03 (95% CI 0.94 to 1.12) | 9.2 | 0.354 |
| Any adverse events | Daprodustat | RR | 4 | 1.03 (95% CI 1.00 to 1.07) | 0.0 | 0.615 |
| Any adverse events | Desidustat | RR | 2 | 0.98 (95% CI 0.86 to 1.12) | 0.0 | 0.552 |
| Any adverse events | Roxadustat | RR | 10 | 1.01 (95% CI 1.00 to 1.03) | 0.0 | 0.774 |
| Hyperkalemia | Roxadustat | RR | 5 | 1.21 (95% CI 0.86 to 1.70) | 37.0 | 0.175 |
| Change in ferritin from baseline | Roxadustat | SMD | 6 | -0.10 (95% CI -0.24 to 0.04) | 38.8 | 0.147 |
| Monthly intravenous iron dose | Roxadustat | SMD | 2 | -0.25 (95% CI -0.34 to -0.16) | 0.0 | 0.786 |
| All-cause mortality | Daprodustat | RR | 4 | 1.00 (95% CI 0.85 to 1.17) | 0.0 | 0.592 |
| All-cause mortality | Desidustat | RR | 2 | 0.77 (95% CI 0.34 to 1.76) | 0.0 | 0.506 |
| All-cause mortality | Molidustat | RR | 2 | 3.54 (95% CI 0.58 to 21.43) | 0.0 | 0.780 |
| All-cause mortality | Roxadustat | RR | 7 | 1.06 (95% CI 0.94 to 1.20) | 9.3 | 0.358 |
| All-cause mortality | Vadadustat | RR | 3 | 1.61 (95% CI 0.85 to 3.04) | 0.0 | 0.783 |
| Serious adverse events | Daprodustat | RR | 3 | 1.19 (95% CI 1.10 to 1.28) | 0.0 | 0.413 |
| Serious adverse events | Desidustat | RR | 2 | 1.02 (95% CI 0.59 to 1.76) | 39.2 | 0.200 |
| Serious adverse events | Roxadustat | RR | 8 | 1.05 (95% CI 1.00 to 1.09) | 0.0 | 0.801 |
| Serious adverse events | Vadadustat | RR | 4 | 0.94 (95% CI 0.89 to 1.00) | 0.0 | 0.757 |

eTable 7. Supplementary pooled estimates for secondary and safety outcomes

| **Outcome** | **Effect** | **Number of studies (k)** | **Random-effects model (95% CI)** | **Fixed-effects model (95% CI)** | **Heterogeneity (I², %)** | **Cochran's Q p-value** |
| --- | --- | --- | --- | --- | --- | --- |
| Change in hemoglobin from baseline | MD | 9 | 0.99 (95% CI 0.45 to 1.52) | 0.71 (95% CI 0.65 to 0.76) | 98.7 | <0.001 |
| Hemoglobin level during treatment | MD | 3 | -0.27 (95% CI -0.56 to 0.02) | -0.20 (95% CI -0.32 to -0.08) | 79.7 | 0.007 |
| MACE / cardiovascular events | RR | 17 | 1.01 (95% CI 0.95 to 1.06) | 1.01 (95% CI 0.95 to 1.06) | 0.0 | 0.730 |
| Any adverse events | RR | 18 | 1.02 (95% CI 1.00 to 1.03) | 1.02 (95% CI 1.00 to 1.03) | 0.0 | 0.914 |
| Hyperkalemia | RR | 5 | 1.21 (95% CI 0.86 to 1.70) | 1.20 (95% CI 0.95 to 1.52) | 37.0 | 0.175 |
| Change in ferritin from baseline | SMD | 7 | -0.14 (95% CI -0.28 to 0.01) | -0.08 (95% CI -0.17 to 0.01) | 42.7 | 0.106 |
| All-cause mortality | RR | 18 | 1.05 (95% CI 0.96 to 1.14) | 1.05 (95% CI 0.96 to 1.14) | 0.0 | 0.665 |
| Serious adverse events | RR | 19 | 1.04 (95% CI 0.99 to 1.10) | 1.03 (95% CI 1.00 to 1.07) | 47.3 | 0.012 |

eTable 8. Sensitivity analyses restricted to studies at low risk of bias

| **Outcome** | **Effect** | **k** | **Random-effects (95% CI)** | **Fixed-effect model (95% CI)** | **Heterogeneity (I², %)** | **Cochran's Q p-value** |
| --- | --- | --- | --- | --- | --- | --- |
| Any intravenous iron use | RR | 0 | No low-risk studies | NA | NA | NA |
| Any red blood cell transfusion | RR | 0 | No low-risk studies | NA | NA | NA |
| Monthly intravenous iron dose | SMD | 0 | No low-risk studies | NA | NA | NA |
| Rescue red blood cell transfusion | RR | 3 | 0.40 (95% CI 0.16 to 0.99) | 0.54 (95% CI 0.46 to 0.64) | 23.9 | 0.269 |

eTable 9. Summary of leave-one-out sensitivity analyses

| **Outcome** | **Effect** | **Direction/significance after leave-one-out** | **Interpretation for readers** |
| --- | --- | --- | --- |
| Any red blood cell transfusion | RR | Direction unchanged; statistical significance was largely retained. | The reduction in transfusion risk was generally robust. |
| Rescue red blood cell transfusion | RR | Direction unchanged; significance became borderline or non-significant after selected omissions. | Benefit was directionally consistent but study-sensitive. |
| Any intravenous iron use | RR | Direction unchanged; statistical significance was unstable. | Suggests reduced IV iron use, but the estimate was less robust. |
| Monthly intravenous iron dose | SMD | Direction unchanged; statistical significance was unstable. | Direction favored lower IV iron dose; interpret cautiously. |
| MACE / cardiovascular events | RR | No material change; estimates remained close to the null. | Cardiovascular safety findings were stable. |
| All-cause mortality | RR | No material change; remained non-significant. | Mortality findings were not driven by a single study. |
| Serious adverse events | RR | Direction unchanged; statistical significance was sensitive to selected omissions. | SAE estimates were close to null and should be interpreted cautiously. |

eTable 10. Descriptive summary of continuous outcomes with substantial heterogeneity

| **Outcome** | **Effect** | **Number of studies (k)** | **Random-effects model (95% CI)** | **Heterogeneity (I², %)** | **Cochran's Q p-value** |
| --- | --- | --- | --- | --- | --- |
| Monthly intravenous iron dose | SMD | 5 | -0.19 (95% CI -0.42 to 0.03) | 89.2 | <0.001 |
| Change in hemoglobin from baseline | MD | 9 | 0.99 (95% CI 0.45 to 1.52) | 98.7 | <0.001 |
| Hemoglobin level during treatment | MD | 3 | -0.27 (95% CI -0.56 to 0.02) | 79.7 | 0.007 |

eTable 11. GRADE evidence profile

| **Outcome** | **Risk of bias** | **Inconsistency** | **Indirectness** | **Imprecision** | **Publication bias** | **Overall certainty** |
| --- | --- | --- | --- | --- | --- | --- |
| Any red blood cell transfusion | Not downgraded | Downgraded 1 | Not downgraded | Not downgraded | Not detected | Moderate |
| Rescue red blood cell transfusion | Not downgraded | Downgraded 1 | Not downgraded | Not downgraded | Not detected | Moderate |
| Any intravenous iron use | Downgraded 1 | Downgraded 1 | Not downgraded | Not downgraded | Not detected | Low |
| Monthly intravenous iron dose | Downgraded 1 | Downgraded 1 | Not downgraded | Not downgraded | Not detected | Low |
| Major adverse cardiovascular events (MACE) | Not downgraded | Not downgraded | Not downgraded | Not downgraded | Not detected | Moderate |
| Serious adverse events | Not downgraded | Not downgraded | Not downgraded | Not downgraded | Not detected | Moderate |
| Mortality | Not downgraded | Not downgraded | Not downgraded | Not downgraded | Not detected | Moderate |
| Change in hemoglobin from baseline | Downgraded 1 | Downgraded 2 | Not downgraded | Not downgraded | Not detected | Low |

eFigure 1. Forest plot of any red blood cell transfusion stratified by HIF-PHI

eFigure 2. Forest plot of rescue red blood cell transfusion stratified by HIF-PHI

eFigure 3. Forest plot of mean monthly intravenous iron dose

eFigure 4. Forest plot of any intravenous iron use stratified by HIF-PHI

eFigure 5. Forest plot of mean monthly intravenous iron dose stratified by HIF-PHI

eFigure 6. Forest plot of change in hemoglobin from baseline

eFigure 7. Forest plot of hemoglobin level during treatment

eFigure 8. Forest plot of change in ferritin from baseline

eFigure 9. Forest plot of any adverse event

eFigure 10. Forest plot of hyperkalemia

eFigure 11. Funnel plot for any red blood cell transfusion

eFigure 12. Funnel plot for major adverse cardiovascular events

eFigure 13. Funnel plot for any adverse event

eFigure 14. Funnel plot for all-cause mortality

eFigure 15. Funnel plot for serious adverse events
